# The Sindbis virus nsP3 opal codon protects viral RNA and fitness by maintaining replication spherule integrity

**DOI:** 10.1101/2025.09.27.679005

**Published:** 2025-10-06

**Authors:** Tamanash Bhattacharya, Tiia S. Freeman, Eva M. Alleman, Fang Wang, Lyuba Chechik, Michael Emerman, Kevin M. Myles, Harmit S. Malik

**Affiliations:** a Basic Sciences Division, Fred Hutchinson Cancer Center, Seattle, WA, USA; b Department of Entomology and AgriLife Research, Texas A&M University, College Station, TX, USA; c Human Biology Division, Fred Hutchinson Cancer Center, Seattle, WA, USA; d Howard Hughes Medical Institute, Fred Hutchinson Cancer Center, Seattle, WA, USA

**Keywords:** alphaviruses, opal codon, host-switching, RNAi, mosquito

## Abstract

Most alphaviruses encode an in-frame opal stop codon between nsP3 and nsP4 in their non-structural ORF. This opal stop codon mediates a temperature-dependent balance between viral polymerase production and proteolytic processing in vertebrate hosts. Yet, why this opal codon is maintained in insect hosts is unknown. Here, we show that the nsP3 opal stop codon confers a replicative advantage to Sindbis virus (SINV) in RNAi-competent mosquito cells, but not in cells lacking RNAi. Through delays in nsP processing, the lack of opal stop codon disrupts viral replication spherule integrity and increases Dicer 2-dependent cleavage of viral RNA, resulting in higher antiviral siRNA responses to the virus. Moreover, in mammalian cells, the opal codon-mediated spherule integrity also blocks MDA5-dependent viral RNA detection and interferon signaling. Thus, the highly conserved alphavirus opal codon mediates a multipotent viral defensive strategy.

## Introduction

**Figure 1.**
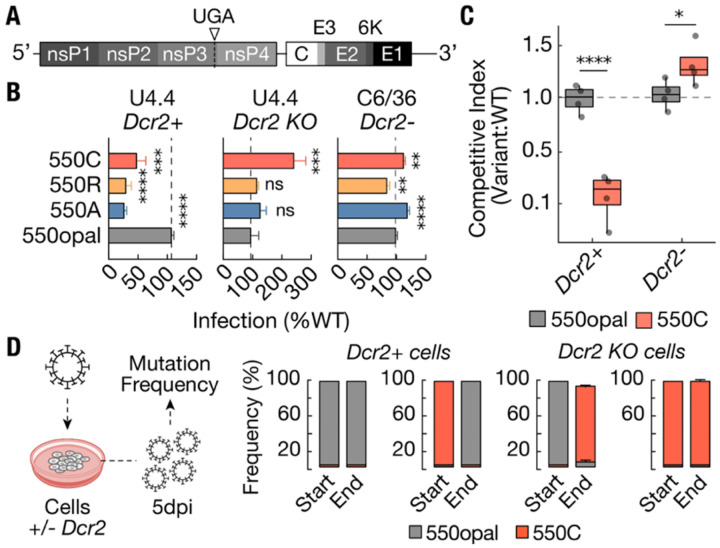
The mosquito RNAi pathway imposes strong selection on the alphavirus nsP3/4 opal codon. (A) Alphaviruses encode a premature in-frame opal codon within the non-structural open reading frame at the 3’ end of the nsP3 gene. (B) Infection rates of opal-to-sense substitution SINV variants relative to wild-type SINV in *Aedes albopictus* cells: either U4.4 cells with functional *Dcr2 (Dcr2+)* or CRISPR/Cas9-edited Ocr2-deficient *(Dcr2* KO) U4.4 cells, or *(Dcr2*−) C6/36 cells. Data represent the mean of at least three independent biological replicates. (C) Competitive fitness of SINV 550C variant against wild-type SINV 550opal in *Dcr2+* U4.4 or *Dcr2*− C6/36 cells. Wild-type versus wild-type competition was performed as a control. The data represent four independent biological replicates. Error bars represent the standard error of the mean (SEM). Two-way ANOVA with Tukey’s test for multiple comparisons. **** = P < 0.0001, *** = P < 0.001, ** = P < 0.01, * = P < 0.05, ns = not significant. (D) Wild-type SINV (with opal codons, UGA) or 550C SINV variants (with UGC/UGU codons) were passaged for 5 days in *Dcr2+* U4.4 cells or CRISPR-edited U4.4 *Dcr2* KO cells. The frequencies of opal versus cysteine codons at the start and end of the passaging, as determined via small RNA sequencing, are reported. The data represent three independent biological replicates.

Arboviruses encounter markedly different cellular environments during their alternating transmission between insect vectors and vertebrate hosts, requiring various strategies to adapt to the contrasting temperatures and immune responses of each host. A key adaptation that allows dual-host alphaviruses to cope with these environmental changes is an in-frame premature opal (UGA) stop codon within the non-structural polyprotein (nsP) open reading frame at the end of the *non-structural protein 3 (nsP3)* gene, just before the *non-structural protein 4 (nsP4)* gene (Figure 1A). Most dual-host alphaviruses retain this *nsp3* opal stop codon (1). The opal codon is part of a Type-II programmed ribosomal readthrough (PRT) system, which features a UGA stop codon followed by cytosine, with uracil bases preferred at positions -2 and -3 (2–4). When the ribosome encounters the opal stop codon, cellular tRNAs outcompete normal translation termination factors like eRF1 to insert an amino acid at a frequency of 10–15% instead of stopping translation (1, 5, 6). This process enables alphaviruses to produce two different polyproteins from their *nsP* genes: the standard translation produces the nsP1-nsP2-nsP3 polyprotein (P123), whereas PRT results in the longer nsP1-nsP2-nsP3-nsP4 polyprotein (P1234) (7). Because nsP4 functions as the viral RNA polymerase necessary for generating new viral RNA, sufficient readthrough is vital for viral replication (4).

There has been considerable interest in understanding how and why the opal codon provides a fitness advantage to dual-host alphaviruses (8–11). We recently demonstrated that alphaviruses use the opal stop codon to resolve a temperature-dependent trade-off between nsP4 expression via translational readthrough and the proteolytic processing efficiency of the viral nsP (1). In vertebrate cells that grow at 37°C, the opal codon is strongly preferred over non-opal substitutions at this site, whereas non-opal (sense codon) substitutions are much better tolerated in vertebrate cells at 28°C or *Aedes albopictus*-derived C6/36 mosquito cells at 28°C (1, 11, 12). Previous studies with distinct alphaviruses, including SINV and Eastern equine encephalitis virus (EEEV), showed that Opal-to-Cysteine substitutions may even provide a replicative fitness advantage over the wild-type opal codon in C6/36 cells (1, 13). Despite this apparent fitness benefit, opal-to-sense codon substitutions are exceedingly rare (3%) in alphaviruses isolated from mosquitoes and are entirely absent in closely related insect-specific alphaviruses (Figure S1B) (14–16). Thus, additional host or environmental factors must contribute to the conservation of the nsP3 opal stop codon in insect hosts.

Previous studies have demonstrated that alphaviruses elicit a robust small RNA response in mosquito cells, mediated primarily through the siRNA pathway (17–19). C6/36 cells lack an effective antiviral RNA interference (RNAi) response due to impaired function of Dicer 2 (*Dcr2*), a key mediator in the short interfering RNA (siRNA) pathway (20). Following viral infection, *Dcr2* processes viral double-stranded RNA (dsRNA) replication intermediates into 21-nt-long virus-derived small interfering RNAs (vsiRNAs). These in turn form a complex with Argonaute 2 (Ago2) and other components of the RNA-induced silencing complex (RISC) to target nascent single-stranded viral RNA for cleavage and degradation (21, 22). Here, we investigated whether immune pressures from RNAi might explain the retention of the opal codon in insect hosts using the prototype alphavirus Sindbis virus (SINV) as our model. We found that an Opal-to-Cysteine substitution (SINV 550C) enables SINV to replicate better than wild-type SINV (550opal) in *Aedes albopictus*-derived RNAi-deficient C6/36 cells or in U4.4 cells depleted of key RNAi proteins. However, the SINV 550C variant is significantly impaired in fitness relative to wild-type SINV (550opal) in RNAi-competent U4.4 cells. We demonstrate that the integrity of intracellular viral replication compartments, or spherules, is compromised in SINV 550C, but not 550opal, due to delays in nsP processing. This lack of spherule integrity does not impair fitness in the absence of RNAi. However, in RNAi-competent mosquito cells, the lack of spherule integrity enables host antiviral defenses, such as *Dcr2*, to access viral dsRNA and induce a significantly higher antiviral siRNA response, thereby lowering the fitness of the SINV 550C variant. Thus, the opal codon is likely retained in insect alphaviruses to protect against the RNAi immune response. We also demonstrate that SINV and other alphaviruses utilize structured RNA elements within their genomes to generate “decoy” vsiRNAs, which blunt the mosquito RNAi response when spherule integrity is compromised. We further demonstrate that compromised spherule integrity also triggers increased viral RNA detection by RNA sensors in vertebrate cells, leading to SINV 550C infections producing a more robust antiviral interferon response. Thus, by ensuring timely polyprotein processing, the opal codon helps maintain spherule integrity that limits access to host cytoplasmic proteins that detect or cleave viral dsRNA in both insect and vertebrate hosts. Collectively, these findings suggest that mosquito-transmitted alphaviruses maintain the opal stop codon as a defensive strategy.

## Results

### The Dcr2-dependent siRNA pathway imposes selection against non-opal codon variants

The *Dcr2*-dependent siRNA pathway serves as the primary antiviral response against alphavirus infections in mosquitoes (17). *Aedes albopictus*-derived C6/36 cells lack functional *Dcr2* due to a frameshift mutation within the *dcr-2* open reading frame, resulting in a Dicer 2 protein that lacks the RNase III domains essential for dicing activity (20). To investigate whether *Dcr2* deficiency explains the tolerance of opal codon substitution variants in C6/36 cells, we compared the growth of three opal-to-sense substitution variants (550Alanine, 550Arginine, 550Cysteine) in *Dcr2*-competent U4.4 cells or *Dcr2*-lacking C6/36 cells. To ensure quantifiable virus growth in RNAi-competent cells, all infections were performed at moderately high MOI (≥4). Consistent with our previous study (1), we found that infection rates of 550C and 550A variants were higher than wild-type SINV (550opal) in *Dcr2*-lacking C6/36 cells, whereas the SINV 550R was less fit. In contrast, infection rates of all sense-codon variants were significantly reduced in *Dcr2*-competent U4.4 cells compared to wild-type SINV (550opal) (Figure 1B).

Both U4.4 and C6/36 cells are derived from *Aedes albopictus*. Nevertheless, differences between these cell lines, beyond their *Dcr2* status, could influence SINV infections. To compare the fitness of viral variants within isogenic host cell lines, we used CRISPR/Cas9 to create *dcr-2* knockout (*Dcr2* KO) U4.4 cells, resulting in a cell population in which *Dcr2* was knocked out in ~82–85% of the cells (Figure S2A). Despite the incomplete nature of this knockout, we found that infection rates of all SINV opal-to-sense variants, including SINV 550R, were restored (550A, 550R) or improved (550C) relative to wild-type SINV (550opal) in U4.4 *Dcr2* KO cells (Figure 1B). Thus, in both C6/36 and U4.4 cells, loss of *Dcr2* enhances the fitness of SINV opal-to-sense variants.

Our recent comprehensive profiling of the SINV opal codon in *Dcr2*-deficient mosquito cells showed that cysteine is the most tolerated sense codon (1). Cysteine is also the only opal-to-sense substitution observed in SINV in nature (23). We therefore carried out all subsequent comparative analyses using wild-type SINV and SINV 550C. In agreement with the independent growth assays, our competition experiments revealed that SINV 550C fitness is significantly reduced relative to 550opal in *Dcr2*-competent U4.4 cells, whereas it is higher than wild-type SINV in *Dcr2*-lacking C6/36 cells (Figure 1C), recapitulating our previous findings (1).

We also evaluated the selective pressure imposed by intact *Dcr2* by passaging SINV 550C variants across three independent replicate lineages during a five-day infection, *i.e.,* approximately 24 rounds of replication (Figure 1D). We found that SINV550C was almost entirely replaced by 550opal in *Dcr2*-competent U4.4 cells, implying that mutations reverting 550C to 550opal had occurred and swept through the population within just 24 rounds of replication. In contrast, we found that 550C variants were still maintained in U4.4 *Dcr2* KO cells (Figure 1D). Conversely, when we passaged 550opal variants in U4.4 *Dcr2* KO cells, we found that they almost completely converted to 550sense variants, primarily 550C (UGC or UGU codons), within the same time frame of 5 days. Yet, 550opal variants were still maintained upon passaging in *Dcr2*-competent U4.4 cells (Figure 1D). The rapid gain of opal-to-sense substitutions in *Dcr2* KO U4.4 cells mirrors prior findings in Eastern equine encephalitis virus (EEEV), which also acquire opal-to-sense mutations following passaging in *Dcr2*-deficient C6/36 cells (13). Together, our findings demonstrate that *Aedes Dcr2* imposes an intense selective pressure to retain the SINV opal codon (Figure 1).

**Figure 2.**
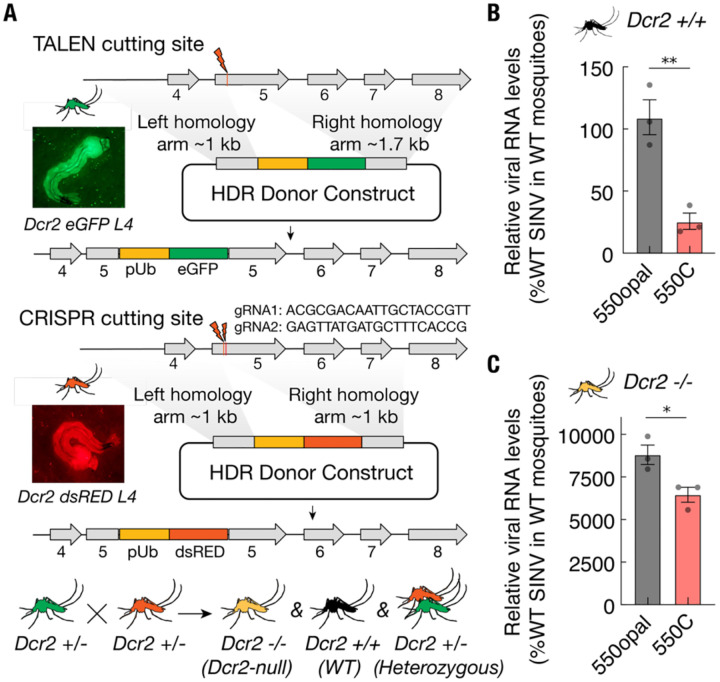
The mosquito RNAi pathway imposes strong selection on SINV nsP3 opal codon *in vivo*. (A) (Top) Schematic of TALEN-mediated HOR integration to generate Ocr2-null transgenic lines with the knock-in of a frame-disrupting pUb-eGFP marker. (Middle) Schematic of CRISPR-Cas9-mediated Ocr2-null transgenic lines with the knock-in of a frame-disrupting pUb-dsRED marker. Photographs are representative of the trans-heterozygous eGFP or dsRED-expressing mosquitoes selected for crossing experiments (Bottom) to generate wild-type *(Dcr2+/+,* black) or homozygous Ocr2-null *(Dcr2−/−,* yellow) mosquitoes. (B-C) Wild-type *(Dcr2+/+)* or Ocr2-null *(Dcr2−/−) Aedes aegypti* mosquitoes were infected with SINV 550opal or 550C. Four days post-infection, viral RNA was quantified via qRT-PCR. The data represent three independent biological replicates, each consisting of four pooled mosquitoes. Error bars represent the standard error of the mean (SEM). Unpaired t-test. ** = P < 0.01, * = P < 0.05.

We next tested whether the *in vitro* fitness differences between SINV 550opal and SINV 550C translated into altered infection outcomes in *Aedes* mosquitoes. For this, we used two reagents. The first is a previously published *Aedes aegypti Dcr2* loss-of-function mutant generated by TALEN, in which a constitutively expressed eGFP knock-in cassette is used to knock out *Dcr2* (ref). Second, we generated a CRISPR/Cas9-mediated *Dcr2* knock-out line with a constitutively expressed *dsRED* inserted into *Dcr2*, thereby disrupting the open reading frame (Figures 2A) (24). Trans-heterozygous expression of eGFP and dsRED enables us to visually track the independent insertions in loss-of-function mutants. By crossing the independent heterozygous *A. aegypti* lines, we were able to generate trans-heterozygous knockout of *Dcr2* (yellow, Figure 2A). We then infected wild-type or *Dcr2−/−* knockout sibling *A. aegypti* mosquitoes with either wild-type SINV (550opal) or the 550C SINV variant. Consistent with our *in vitro* findings, we found that viral RNA levels in SINV 550C-infected wild-type mosquitoes were 4-fold lower relative to those infected with SINV 550opal four days post-infection (Figure 2B). This implies that SINV 550C incurs a significant loss in viral dissemination in wild-type mosquitoes. As expected, the loss of *Dcr2* significantly improved infection levels for both wild-type SINV (550opal) and SINV 550C. Although SINV 550C is still less fit than wild-type SINV (550opal) in *Dcr2−/−* mosquitoes, its relative fitness loss is significantly ameliorated in the absence of host *Dcr2* (Two-way ANOVA, 550C x*Dcr2* Interaction: p = 0.0134); SINV 550C RNA levels were only 1.5-fold lower than SINV 550opal (Figure 2C). These findings confirm that the loss of RNAi partially rescues replication of the SINV 550C variant *in vivo* (25, 26).

### Opal-to-Cys substitution induces a higher small RNA response in Dicer 2-competent mosquito cells

**Figure 3.**
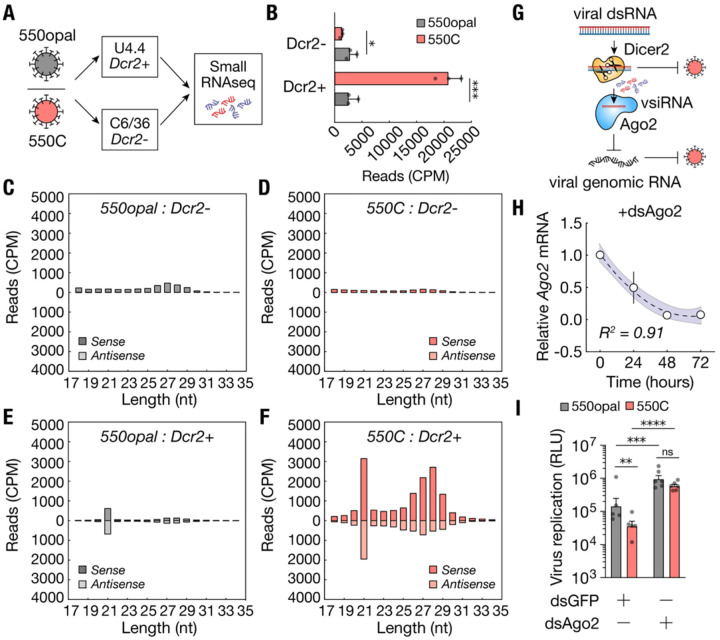
SINV sense-codon variant induces higher small RNA response in mosquito cells. (A) *Dcr2+* U4.4 or *Ocr2-* C6/36 cells were infected with wild-type SINV 550opal or the SINV 550C variant. Small RNA sequencing was performed on cells collected five days post-infection. (8) Normalized small RNA read counts mapped to SINV genomes in *Ocr2+* U4.4 or *Dcr2*− C6/36 cells infected with wild-type SINV 550opal and SINV 550C. (C-D) Size distribution of small RNAs derived from *Dcr2*− C6/36 cells infected with wild-type SINV 550opal or SINV 550C. (E-F) Size distribution of small RNAs derived from *Ocr2+* U4.4 cells infected with wild-type SINV 550opal or SINV 550C. The data represent three independent biological replicates. (G) Schematic of mosquito RNAi pathway and the roles of Dcr2 and Ago2 in antiviral restriction. (H) Quantification of relative *Ago2* transcript levels by qRT-PCR in *Ocr2+* mosquito cells treated with *Ago2* double- stranded RNA (dsAgo2). The dotted line denotes the expected trend from a one-phase exponential decay model. Bands represent the 95% confidence interval. (I) Effect of dsAgo2 treatment on the replication of wild-type SINV 550opal and SINV 550C in *Ocr2+* mosquito cells as quantified by luciferase assay. Error bars represent the standard error of the mean (SEM). Student’s t-tests. *** = P < 0.001, * = P < 0.05, ns = not significant.

Since SINV 550C exhibits reduced fitness in *Dcr2*-competent cells, we hypothesized that this variant might induce a more pronounced siRNA response compared to wild-type SINV (550opal). To test this hypothesis, we performed small RNA sequencing analysis of *Dcr2*+ U4.4 or *Dcr2*− C6/36 *Aedes albopictus* cells infected with either 550opal or 550C SINV variants (Figure 3A). *Dcr2 KO* U4.4 cells were not chosen for small RNA sequencing analysis due to the incomplete nature of the knockout. Although SINV 550C infected nearly 10-fold fewer cells compared to wild-type SINV (Figure S4A), virus-derived small RNA levels were significantly elevated in *Dcr2*+ cells infected with SINV 550C relative to those infected with wild-type SINV (550opal) (Figure 3B). Thus, a single nucleotide polymorphism (UGA to UGC) in the 11.7 kb SINV genome is sufficient to trigger a substantially higher small RNA response in mosquito cells.

To further dissect the exact nature of the small RNA response, we analyzed small RNAs based on their size distribution and polarity (Figure S4B–E). As expected, the siRNA response against either SINV 550C or SINV 550opal was markedly attenuated in C6/36 cells that lack *Dcr2* (Figures 3C–D). Moreover, all the small RNAs in C6/36 cells appear to be biased towards the sense strand. However, in *Dcr2*-competent U4.4 cells, levels of 21-nucleotide vsiRNAs were substantially elevated in SINV 550C-infected cells compared to 550opal-infected cells (Figures 3E–F), consistent with SINV 550C’s enhanced susceptibility to *Dcr2*-mediated processing (Figures 3B). We also observed distinct differences in the polarity of siRNAs generated from the two viral variants; siRNAs derived from 550opal mapped equally to sense and antisense viral RNA, suggesting they originate from double-stranded RNA replication intermediates, in which sense and antisense RNAs are present at equimolar ratios (Figure 3E). In contrast, 550C-derived siRNAs exhibited a strong bias toward the sense strand (Figure 3F). Our findings indicate that siRNAs generated in SINV 550C variant-infected cells are not only more abundant than those generated in wild-type SINV (550opal)-infected cells but also differ in that a significant fraction of siRNAs originates from single-stranded positive-sense viral RNA instead of double-stranded RNA.

The SINV 550C variant produces substantially more siRNAs and has lower fitness than the wild-type SINV (550opal) in *Dcr2*-expressing U4.4 cells. We considered two complementary mechanisms by which higher siRNA production could directly lead to lower fitness (Figure 3G, top). First, siRNA biogenesis from *Dcr2*-mediated cleavage of viral dsRNA could be inherently damaging to the virus due to the loss of minus-strand replication template. This would result in a stoichiometric reduction of intact viral dsRNA, which by itself might be sufficient to lower viral fitness. Second, viral siRNAs generated through *Dcr2* activity can be subsequently targeted to degrade nascent viral RNA via Argonaute 2 (Ago2), resulting in downstream restriction (Figure 3G, bottom). To distinguish between these two mechanisms, we tested whether Ago2-mediated targeting of vsiRNAs to viral RNAs is necessary for SINV 550C inhibition. To achieve this, we performed an *Ago2* knockdown using double-stranded RNA (dsRNA) against *Ago2* in U4.4 cells, resulting in greater than 90% knockdown within 48 hours (Figure 3G–H, Figure S5A). If both cleavage and Ago2 targeting played substantial antiviral roles, then we would expect that an *Ago2* knockdown would have had only a modest impact on viral fitness (Figure 3G). In contrast to this expectation, we found that an *Ago2* knockdown substantially increased the replication of the SINV 550C variant to levels that were indistinguishable from those of wild-type SINV (550opal) (Figure 3I). This result demonstrates that, instead of viral dsRNA loss due to initial *Dcr2*-mediated dsRNA processing, it is the subsequent Ago2-mediated vsiRNA targeting of viral RNA that substantially contributes to the loss of SINV 550C fitness in *Dcr2*+ cells.

In addition to vsiRNAs, we also found viral-derived 24–31nt piwi-interacting RNAs (piRNAs) in both C6/36 and U4.4 cells, similar to previous studies (Figures 3C–F, S6, S7, S8A) (18, 19, 27, 28). In mosquito cells, viral piRNAs (vpiRNAs) are predominantly generated via a ping-pong-like mechanism that utilizes specific PIWI proteins, such as Ago3 and Piwi5 (29). Consistent with previous studies, we found that SINV-derived piRNA reads originated primarily from the subgenomic RNA, particularly from a hotspot at the 5’ end of the capsid gene (Figure S6B) via a canonical ping-pong amplification pathway (Figure S8C) (28, 30). Just like siRNAs, we found that C6/36 cells accumulate significantly fewer viral-derived piRNAs than U4.4 cells (Figures 3C–F, S8A–B). *Dcr2*+ U4.4 cells infected with the SINV 550C variant also accumulate 24–31nt long viral-derived piRNAs at 12-fold higher levels than the wild-type SINV variant (Figures 3B–C and S8A). In contrast, SINV 550C infection is associated with a 2-fold reduction in small RNAs in mosquito cells lacking *Dcr2*, which exhibit a near-complete lack of vsiRNA and vpiRNA production (Figure 3E–F). Thus, even though vpiRNA production is canonically thought to be independent of mosquito *Dcr2* activity, our results add to a growing body of evidence that suggests a functional crosstalk between the siRNA pathway and the production of ping-pong generated piRNAs in mosquitoes (27, 31–33). Unlike viral-derived siRNAs, which have been previously shown to restrict alphavirus replication in insect cells, the antiviral role of viral-derived piRNAs is more ambiguous (30, 31, 34). In our study, we did not observe any significant changes in levels of the subgenomic RNA template or structural protein expression between wild-type SINV (550opal) and SINV 550C-infected *Dcr2*+ cells (Figure S8D). Moreover, since knockdown of *Ago2*, which is involved in siRNA but not piRNA targeting, was sufficient to restore the fitness of the SINV 550C variant to wild-type levels in U4.4 cells (Figure 3I), we conclude that viral-derived piRNAs do not play a substantial antiviral role and do not discuss them further in this study.

### SINV Structured RNA elements defend against RNAi

**Figure 4.**
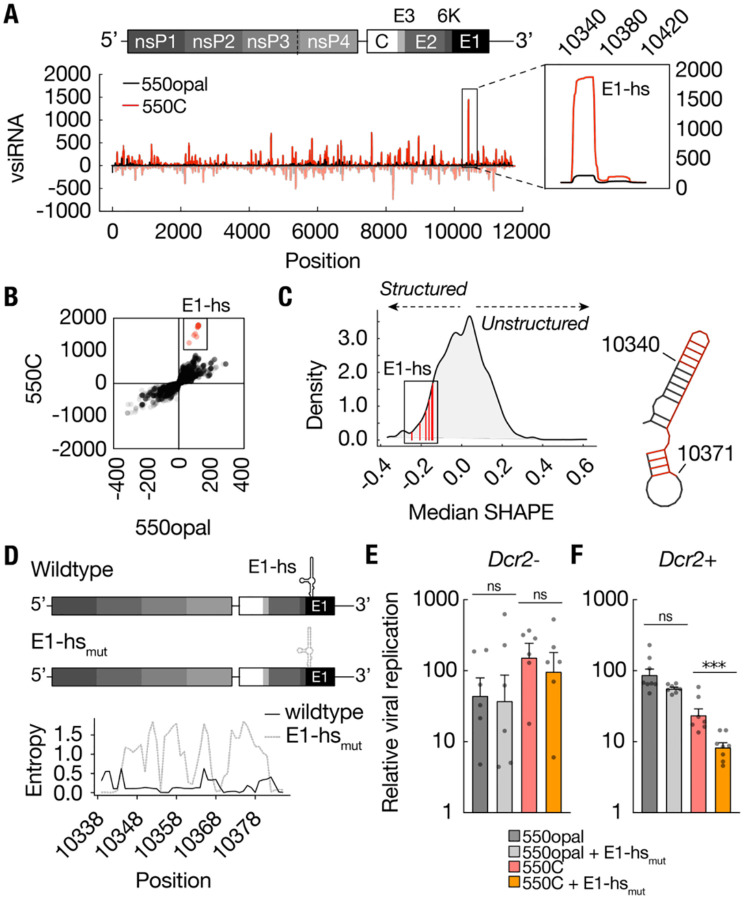
A structured RNA element enables SINV to blunt the siRNA response. (A) Distribution of vsiRNA reads from wildtype SINV 550opal (in black) or SINV 550C (in red) viruses in *Ocr2+* U4.4 cells. Positive and negative Y-axis values represent read counts mapped to the sense and antisense strands, respectively, at every position along the SINV genome (X-axis). The inset shows a zoomed-in view of the E1-hs region. (B) Correlation of per-site vsiRNA reads mapped to wildtype SINV 550opal or SINV 550C viruses. Residues within the E1-hs region are boxed and colored in red. (C) Density distribution profile of median SHAPE values of SINV RNA. Median SHAPE values of residues within the E1-hs region are boxed and highlighted in red. Location of the E1-hs region in SHAPE-constrained RNA secondary structure. (D) Schematic of viral genomes with intact (wildtype) and mutated E1-hs (E1-_hsm_). Entropy profile of E1-hs RNA element in wild-type SINV and the E1-hs_mut_ strain. (E) Replication of wildtype SINV 550opal or SINV 550C viruses with intact or destabilized E1-hs structure in *Dcr2*− C6/36 cells as quantified via luciferase reporter assays. The data represent six independent biological replicates. (F) Replication of wildtype SINV 550opal or SINV 550C viruses with intact or destabilized E1-hs structure in *Dcr2+* U4.4 cells. The data represent six independent biological replicates. Two-way ANOVA with Tukey’s multiple comparisons test. **** = P < 0.0001, *** = P < 0.001, * = P < 0.05, ns = not significant.

To determine whether specific SINV genomic regions act as Dicer 2 substrates in wild-type SINV and particularly SINV 550C-infected cells, we next mapped all 21-nt vsiRNA reads to the SINV reference genome. Using a 40-nucleotide sliding window in the SINV genome, we identified regions with higher-than-average siRNA coverage (Figure 4A). Since infection by the SINV 550C variant elicited a more robust siRNA response than wild-type SINV (550opal), we especially focused on identifying SINV genomic regions that showed disproportionately higher siRNA coverage in SINV 550C compared to wild-type SINV (550opal) (Figure 4B). This differential analysis revealed several windows in the SINV genome that disproportionately generated more siRNAs during infection by the SINV 550C variant. The most striking of these was a 23-nt stretch within the E1 structural gene coding region. This ‘hotspot’, which we designated E1-hs, represented more than 4% of total siRNA reads in SINV 550C (99.85th percentile). We found that E1-hs is also a hotspot in SINV 550opal, producing 1% of total siRNA reads (96th percentile) (Figures 4A–B).

The SINV RNA genome comprises several structural RNA elements essential for its replication, expression, and packaging (35). Due to the biased polarity of vsiRNAs derived from SINV 550C, we were curious whether any such structural RNA elements are hotspots for producing disproportionately large amounts of viral-derived siRNAs. Indeed, the siRNA reads mapping to the E1-hs region exhibited a severe bias toward the sense strand (Figure 4A inset). This suggested that these E1-hs siRNAs may have been derived from some structured RNA element in the SINV genome. To investigate the possibility that E1-hs corresponds to structured RNA, we analyzed previously generated SINV RNA SHAPE-MaP data to calculate and plot the density distribution of median SHAPE reactivity values using the same 40-nucleotide sliding window across the SINV genome (36). Residues within the E1-hs region exhibited distinctly negative SHAPE reactivity values, indicating they are a part of highly structured RNA in SINV (Figure 4C). We assessed the evolutionary conservation of this RNA secondary structure by performing secondary structure prediction using aligned sequences of the E1-hs region from different mosquito-isolated alphaviruses and by referencing prior SHAPE-MaP data collected from Chikungunya virus (CHIKV), which is distantly related to SINV (37). Our analyses revealed a high degree of primary sequence conservation in the E1-hs element (Figure S9). Although some of this conservation may result from constraints on the E1 coding region, we also observed a high degree of covariance between paired residues that form a stem-loop structure, which is more consistent with evolutionary constraints to maintain a structural RNA element. We also compared the genome-wide vsiRNA profiles between SINV 550C and SFV, using data previously collected from SFV4-infected *Dcr2*+ U4.4 *(Aedes albopictus)* and Aag2 *(Aedes aegypti)* cells (19). Our comparison revealed several shared hotspots of vsiRNA production, including the SINV E1-hs region, which were shared between the two viruses, despite their high overall sequence divergence (Figure S10A–B) (19).

To test the functional significance of the E1-hs hotspot, we introduced synonymous mutations designed to disrupt the E1-hs secondary structure without affecting the E1 protein-coding sequence (Figure 4D). We then compared the replication efficiency of these E1-hs_mut_ variants with that of their wild-type counterparts in both SINV 550C and SINV 550opal viruses in *Dcr2*− (C6/36) cells (Figure 4E). In *Dcr2*− cells, we found that the replication of SINV E1-hs_mut_ was indistinguishable from wild-type SINV. Similarly, the replication of SINV 550C E1-hs_mut_ was indistinguishable from SINV 550C. Thus, E1-hs_mut_ variants do not intrinsically impair viral replication. We then investigated the effect of the E1-hs mutation in *Dcr2*+ cells (Figure 4F). We first compared the SINV E1-hs_mut_ variant to wild-type SINV and found that their fitness was not statistically distinct. Next, we compared the replication of SINV 550C E1-hs_mut_ to SINV 550C in *Dcr2*+ cells. We found that the SINV 550C E1-hs_mut_ double mutant replicated to significantly lower levels than SINV 550C (Figure 4F). Thus, the ability of E1-hs to overproduce siRNAs is crucial for the 550C variant in *Dcr2*+ cells, suggesting that the SINV 550C genome might be more susceptible to Dicer 2 targeting. Our findings suggest that the SINV opal codon and the E1-hs structure function synergistically as part of a multipotent RNAi evasion strategy.

### Disrupted nsP processing caused by SINV Opal-to-Cys substitution compromises the integrity of the viral replication spherule, leading to higher susceptibility to RNAi inhibition in mosquito cells

We next asked why the SINV 550C variant is so much more susceptible to RNAi than the wild-type SINV (550opal) variant. We previously found that opal-to-sense substitutions lead to overproduction of the full-length nsP (P1234), which disrupts polyprotein processing kinetics. These disparities in polyprotein processing between wild-type SINV and sense-codon variants are most pronounced in vertebrate cells cultured at 37°C, but also manifest (albeit to a lesser extent) in both vertebrate and mosquito cells maintained at 28°C (1). We therefore considered the possibility that functional RNAi might exacerbate polyprotein processing defects. However, we observed similar polyprotein processing trends in *Dcr2*+ (U4.4) and *Dcr2*− (C6/36) mosquito cells at 28°C (Figure S11A–B), ruling out the possibility that RNAi status affected SINV nsP polyprotein processing. Next, we considered whether the increased RNAi-susceptibility of the SINV 550C variant was due to increased production of viral dsRNA, which could lead to higher production of antiviral siRNAs in *Dcr2*+ cells. This increase in dsRNA could occur as a direct result of the nsP processing defect, which delays transitions from minus-strand to plus-strand replication. We had previously employed a quantitative RT-PCR (qRT-PCR) assay to quantify the intracellular levels of SINV genomic RNA, including both plus and minus strands (1). However, we were concerned that Dcr2 cleavage of viral dsRNA might preclude accurate estimation of viral dsRNA levels within cells. Therefore, to detect both intact and potentially processed dsRNA, we employed the J2 monoclonal antibody, which has been well-characterized to detect both longer and shorter dsRNAs that are 14–40 bp in length (38, 39).

**Figure 5.**
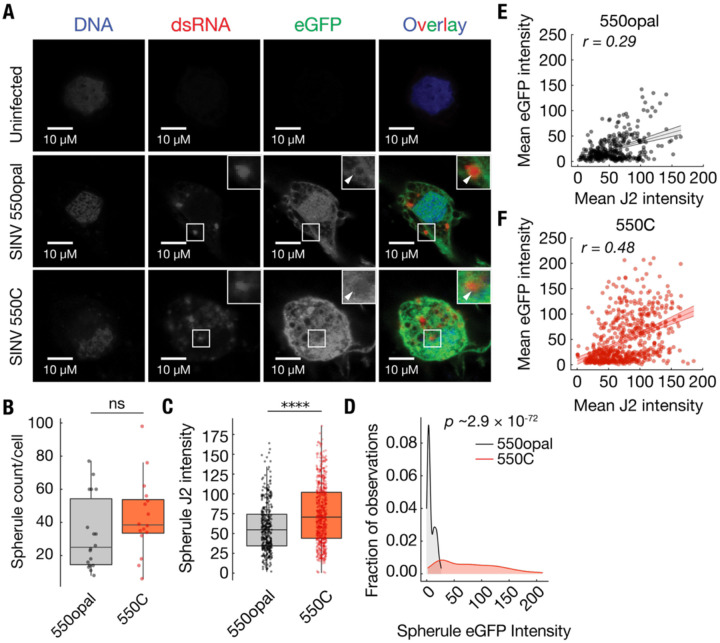
Delayed non-structural polyprotein processing in SINV 550C compromises the integrity of viral replication spherules. (A) Confocal microscopy-based localization of viral dsRNA (J2) and virally encoded eGFP in *Dcr2+* U4.4 cells that were either uninfected (top row), infected with wild-type SINV 550opal (middle row), or infected with SINV 550C (bottom row). The inset shows the localization of eGFP and dsRNA around (middle row) or within (bottom row) viral replication spherules. White arrows indicate spherule outer boundaries. (B) Spherule count per cell is reported as the mean of spherules per cell in each z-stack. (C) Quantification of J2 intensities within viral replication spherules (n=629) in *Dcr2+* U4.4 cells infected with either wild-type SINV 550opal or SINV 550C. Mann-Whitney U test. **** = P < 0.0001, ns = not significant. Error bars represent the standard error of the mean (SEM). (D) Density distributions of observed values of spherule eGFP intensity in wild-type SINV 550opal or SINV 550C-infected *Dcr2+* cells. Kolmogorov-Smirnov (KS) test = 0.862. (E-F) Correlation between mean dsRNA and eGFP intensities per spherule across *Dcr2+* cells infected with either wild-type SINV 550opal or SINV 550C. Spearman’s correlation test (p < 0.0001).

We performed immunofluorescence microscopy on *Dcr2*+ (U4.4) cells, which revealed distinct J2 foci in the cytoplasm of SINV-infected cells, but not in uninfected cells. These J2 foci indicate SINV replication compartments (RCs) or spherules (Figure 5A). We quantified J2/dsRNA intensity within these spherules. While there were no changes in total spherule counts per cell, we found that *Dcr2*+ (U4.4) cells infected with SINV 550C have a 29% increase in spherule J2 intensity compared to those infected with wild-type SINV (550opal), suggesting that SINV 550C replication spherules contain more dsRNA (Figure 5B–C). Recent cryo-EM reconstruction of wild-type alphavirus replication organelles showed that each spherule contains only a single copy of full-length viral dsRNA (40). We therefore wondered whether the accumulation of *Dcr2*-processed dsRNA fragments could lead to an increase in spherule J2 intensity in SINV 550C-infected *Dcr2*+ cells. To test this possibility, we performed a similar analysis in *Dcr2*− C6/36 cells (Figure S12A). We reasoned that if SINV 550C inherently synthesizes excess full-length dsRNA, then we would expect to also see a similar increase in spherule J2 signal in cells lacking *Dcr2*. However, found no significant differences in spherule J2 intensities between wild-type and SINV 550C-infected C6/36 cells (Figure S12B–C). These findings confirm both our previous analyses of greater siRNA production in SINV 550C-infected U4.4 cells and our hypothesis that the increased spherule J2 signal in SINV 550C-infected U4.4 cells (Figure 5B–C) represents an excess of processed dsRNA fragments.

Another consequence of the nsP processing defects in cells infected by the SINV 550C variant could be the compromised integrity of the viral replication spherules, which are comprised of fully processed nsP1, nsP2, and nsP3 proteins. Replication spherules encode a passive but highly effective viral evasion strategy by occluding viral dsRNA replication intermediates from cellular RNA sensors and effectors, including *Dcr2* (41, 42). Presence of processed dsRNA within or proximal to replication spherules in SINV 550C-infected *Dcr2*+ cells suggested a scenario in which mature spherule formation is delayed in SINV 550C due to slower processing of P123 polyprotein, which reduces the structural integrity of replication complexes and allows cytoplasmic *Dcr2* to access and cleave viral dsRNA. To test this hypothesis, we quantified spherule integrity by measuring their ability to occlude virally encoded cytoplasmic eGFP. If viral spherule integrity were maintained, we would expect to see occlusion of the otherwise freely diffusing cytoplasmic eGFP. Indeed, 2D plot profile analysis of dsRNA-containing intact spherules (identified using the J2 antibody) in *Dcr2*+ cells infected with wild-type SINV (550opal) revealed a noticeable decline in eGFP intensity within spherules relative to regions outside the spherule boundary, consistent with the selective exclusion of cytoplasmic eGFP from the viral spherule compartment (Figure S12D). In contrast, within-spherule GFP intensity was noticeably higher in SINV 550C-infected cells. Upon assaying a larger number of J2-marked viral replication spherules in *Dcr2*+ cells infected with wild-type SINV (550opal), we found a bimodal distribution of spherule eGFP intensity values: most spherules exclude eGFP entirely, whereas only a very small subpopulation of spherules cannot exclude eGFP. In contrast, in SINV 550C-infected cells, spherule eGFP intensity values in SINV 550C-infected cells showed a wider unimodal distribution, indicating significantly greater eGFP-leakage into spherules (Figure 5D). Thus, in SINV 550C-infected cells, we found that only a few spherules could exclude eGFP, whereas the vast majority of viral replication spherules could not. We also observed a positive correlation between J2 intensity and eGFP intensity within individual spherules in wild-type SINV-infected *Dcr2*+ cells (Figure 5E). This correlation was stronger in SINV 550C-infected cells, indicating that compromised spherule integrity is associated with higher levels of processed dsRNA within a subset of spherules (Figures 5F).

### Increased susceptibility of SINV Opal-to-Cys variants to cellular RNA sensors and interferon production in vertebrate cells

**Figure 6.**
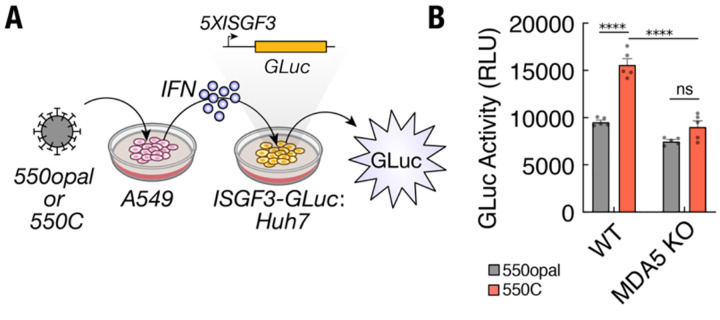
SINV sense-codon variant induces higher IFN response in human cells. (A) A549 cells were infected with either wild-type SINV 550opal or SINV 550C at an MOI of 5. Huh? cells with a 5XISGF3-Gluc reporter were treated for 18h with A549 cell supernatants before the quantification of secreted Gluc. (B) Reporter activity in 5XISGF3-Gluc Huh? cells treated with supernatants collected from wild-type or MDA5 KO A549 cells infected with either wild-type SINV 550opal or SINV 550C at 16 hours post-infection. The data represent the mean of six independent biological replicates. Two-way ANOVA with Sidak’s multiple comparisons test. **** = P < 0.0001, ns = not significant.

Our previous study demonstrated that the nsP processing defects of the SINV 550C variant also occur in vertebrate cells (1). Unlike insects, RNAi is not the predominant immune defense pathway in vertebrate cells. Instead, viral RNA is detected by host-encoded pattern recognition receptors (PRRs) RIG-I and MDA5, which recognize viral dsRNA to trigger downstream interferon (IFN) responses (43). Based on our findings that nsP processing delays result in spherule integrity defects in mosquito cells, we hypothesized that similar spherule defects occur during SINV 550C infection in vertebrate cells. This might trigger dsRNA sensors and differentially induce cellular innate immune pathways. If this were the case, we would expect that SINV 550C-infected cells would trigger a higher interferon response compared to cells infected with wild-type SINV (550opal). We tested this hypothesis by infecting human A549 cells at high MOI with either SINV 550C or SINV 550opal for 16 hours and measured IFN production on Huh7 cells encoding a 5XISGF3-GLuc reporter (Figure 6A) (44). Consistent with our expectation, we observed higher GLuc activity from cells treated with supernatants collected from SINV 550C-infected A549 cells compared to those treated with wild-type SINV (550opal)-infected A549 cell supernatants (Figure 6B).

A549 cells respond to dsRNA by upregulating the expression of RIG-I and MDA5 (45). Prior work has also demonstrated that these PRRs are crucial for IFN induction in response to alphavirus infection (46). We therefore tested whether MDA5, the cytoplasmic RNA sensor implicated in sensing long double-stranded RNA (dsRNA), is responsible for this elevated interferon response in human cells. We infected MDA5 KO A549 cells with either SINV 550C or wild-type SINV (550opal) and measured the resulting interferon response at 16 hours post-infection (hpi). We found that the loss of MDA5 significantly reduced IFN production from SINV 550C-infected A549 cells, confirming that it requires dsRNA sensing by MDA5 (Figure 6B).

**Figure 7.**
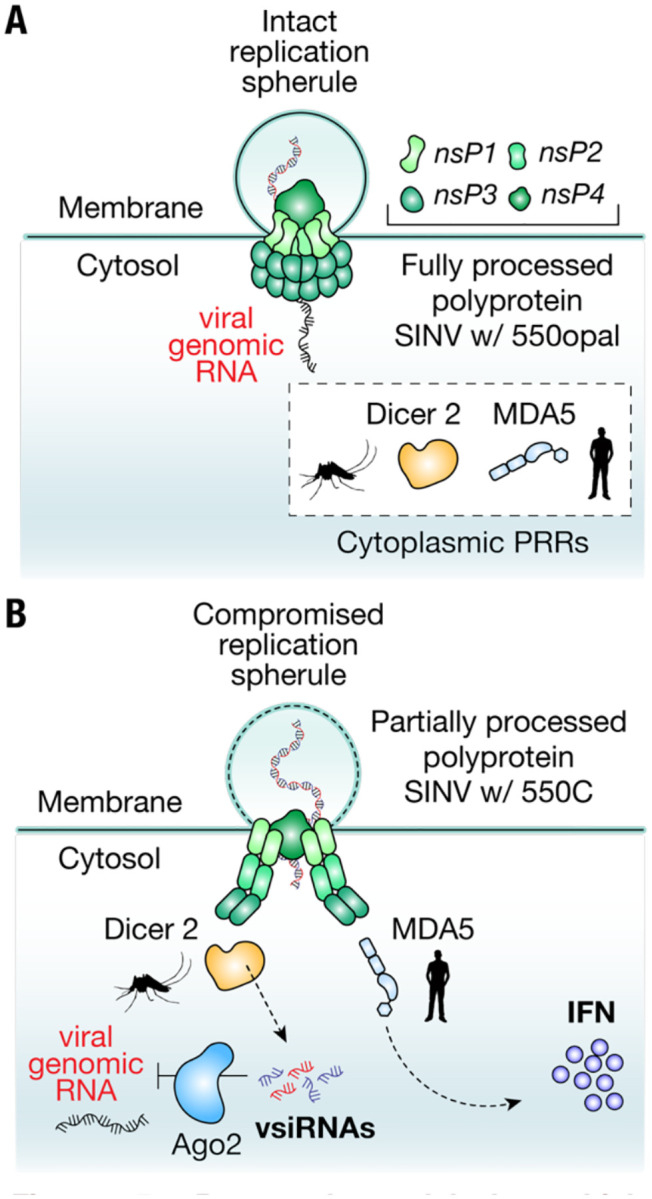
Proposed model by which opal-to-sense codon substitutions lower viral fitness. (A) SINV nsP3 opal codon helps maintain replication spherule integrity to avoid detection by cytoplasmic RNA nucleases (Dcr2) or sensors (MDA5) in mosquito or vertebrate cells, respectively. (B) Improper processing of the nsP polyprotein in SINV 550C variant-infected cells leads to the loss of spherule integrity, resulting in excessive Dcr2 and Ago2-mediated antiviral RNAi induction in mosquito cells and MDA5-mediated IFN induction in vertebrate cells.

Collectively, our data support a model in which delayed nsP processing, caused by Opal-to-Cys substitutions at the stop codon between nsP3 and nsP4, leads to the formation of defective or incompletely formed spherules in alphavirus-infected cells (Figure 7). These structurally compromised spherules enable cytoplasmic proteins, including cytoplasmic RNA sensors such as Dicer 2 in insect cells and MDA5 in vertebrate cells, to access viral dsRNA, leading to enhanced immune activation, ultimately lowering viral fitness in immune-competent insect and vertebrate cells (Figure 7B). Thus, the near-universal conservation of the nsP3 opal codon in alphaviruses is driven by both temperature and defense against host immunity.

## Discussion

Our study demonstrates that the retention of the highly conserved nsP3 opal stop codon is a novel, multipotent defensive strategy employed by alphaviruses (Figure 7A). By systematically dissecting its function in both insect and vertebrate cells, our findings reveal that the opal codon is essential not only for a temperature-dependent trade-off between nsP production and processing, as we previously showed (ref), but also for maintaining the physical integrity of replication spherules that safeguard viral dsRNA from host immune sensors. We find that the opal-to-sense substitution (e.g., SINV 550C) disrupts the cadence of alphavirus non-structural polyprotein processing, leading to the formation of spherules with potentially compromised integrity and consequently increased exposure of viral dsRNA (Figure 7B). This defect enables cytoplasmic Dicer 2 in mosquitoes to access and process viral dsRNA, triggering a much more robust antiviral siRNA response that sharply reduces viral fitness in RNAi-competent *Aedes albopictus* cell lines and in *Aedes aegypti* mosquitoes (Figure 7B). Using small RNA sequencing, we show that SINV 550C, which differs from wild-type SINV by a single nucleotide at the opal codon, induces significantly higher small RNA response in *Aedes albopictus* cells. This direct measurement of RNAi activity in response to wild-type and variant SINV infections provides conclusive evidence for the role of RNAi in the fitness advantage conferred by the opal codon. These findings expand upon findings from earlier work in *Anopheles gambiae*, where an Opal-to-Arginine mutation reduced ONNV infectivity, underscoring the evolutionary importance of preserving the opal codon for efficient vector transmission (8). We also show that alphaviral structural RNA elements, such as E1-hs, protect viral fitness against host RNAi by acting as ‘decoy’ hotspots of vsiRNA production but blunting the antiviral RNAi response. Thus, alphaviruses use a two-pronged defense strategy against mosquito RNAi: first, through physical sequestration of dsRNA within replication spherules, and a secondary mechanism involving the strategic production of ‘decoy’ vsiRNAs from structured genomic RNA elements. Beyond insects, compromised spherule integrity in vertebrate cells may also permit RNA sensors such as MDA5 to detect viral dsRNA, resulting in enhanced interferon signaling (Figure 7B).

Recent *in situ* structural and functional characterization has provided novel insights into the architecture of mature alphavirus replication spherules, which are membrane-associated structures that shield the viral dsRNA from cytoplasmic RNA-sensors in vertebrate and mosquito cells (40, 47–50). While the process of mature spherule biogenesis remains poorly understood, it is thought to require complete proteolytic processing of the nsP into the nsP1, nsP2, and nsP3 proteins that comprise the ‘neck’ of the replication spherule (7, 47, 51). Using quantitative western blotting and confocal microscopy, we show that incomplete polyprotein processing in SINV 550C disrupts the structural integrity of replication spherules, potentially allowing cytoplasmic *Dcr2* access to viral dsRNA in infected mosquito cells. Additionally, the J2 signal intensity in SINV 550C-infected cells is higher than that in cells infected with wild-type SINV (550opal), but only in the presence of *Dcr2*, suggesting that the excess J2 signal may represent processed *Dcr2* substrates, contributing to the overall increase in the siRNA response (Figures 5C, S12B–C). As a result, increased vsiRNA induction by SINV 550C is correlated with slower processing of viral nsP in opal-to-sense substitution variants (Figure S11). However, we cannot distinguish whether higher RNAi induction in SINV 550C-infected mosquito cells is caused by *Dcr2* gaining access to the viral replication compartment or due to viral dsRNA leaking out of the spherules into the host cytoplasm.

Together, these findings suggest that while opal-to-sense substitutions may promote viral replication in *Dcr2*-deficient mosquito cells, the resulting delay in polyprotein processing may compromise the integrity of replication spherules. This structural vulnerability increases viral susceptibility when Dcr2 is present, ultimately limiting the replication efficiency of sense-codon variants in *Dcr2*-competent cells. Our results mirror previous findings of a *Dcr2*-dependent change in the relative replication of CHIKV replicons carrying mutations that delay nsP processing, potentially suggesting a generalizable trade-off between replication efficiency and RNAi susceptibility (52).

Given that RNAi is the primary antiviral defense mechanism in mosquitoes, mosquito-transmitted viruses have evolved several mechanisms to circumvent or suppress the RNAi response while maintaining persistent, non-pathogenic infections in their vectors. For example, non-coding subgenomic flaviviral RNAs (sfRNAs) present at the 3’ end of flavivirus genomes outcompete native *Dcr2* and Ago2 substrates for binding, leading to decreased RNAi activity (53). Other viruses, such as Flock House virus (FHV B2, *Nodaviridae)* and Culex Y virus (CYV VP3, *Birnaviridae),* encode *bona fide* viral suppressors of RNAi (VSRs) (54–56). Alphaviruses are not known to encode sfRNAs or proteins with strong VSR activity, with the possible exception of CHIKV nsP2/3 and SFV capsid, which weakly suppresses RNAi in mammalian cells by potentially sequestering viral dsRNA (57, 58). SINV expressing FHV B2 exhibits high vector mortality and reduced virus transmission (59). Thus, it is likely that, unlike other arboviruses, alphaviruses avoid strong, direct RNAi suppression; they may dampen or evade, rather than completely block, the mosquito RNAi response to maintain persistent nonlethal infection in insect hosts.

Our current understanding of how alphaviruses suppress mosquito RNAi is based on a study using Semliki Forest Virus (SFV), which predominantly lacks the nsP3/4 opal stop codon (19). SFV genomes produce decoy vsiRNAs during infection in mosquito cells that overwhelm the RISC machinery to protect RNAi-susceptible viral targets; however, these decoy vsiRNAs themselves are not antiviral. Additionally, these “decoy” vsiRNAs are derived from genomic hotspots that correspond to structured RNA regions. Consistent with this vsiRNA decoy model, we identified a vsiRNA hotspot in SINV, which maps to a structured RNA element within the E1 coding region (Figures 4A–C). Increased RNA exposure in SINV 550C-infected cells disproportionately affects vsiRNA production from this hotspot element (Figure 4A-inset). Disrupting this RNA secondary structure (E1-hs) significantly reduced SINV 550C replication in RNAi-competent cells, underscoring its role in RNAi evasion (Figures 4D and F). Structural conservation of this RNA element also suggests that it may perform a similar function in other alphaviruses (Figure S9). Although such decoy strategies can be generally beneficial, our findings that mutations in E1-hs specifically lower the fitness of the SINV 550C variant demonstrate that they are especially critical when viruses are more susceptible to Dicer 2 inhibition due to defects in spherule integrity.

Our current results also expand our previous model, in which the nsP3 opal codon helps balance alphavirus polymerase (RdRp) production and nsP processing efficiency in vertebrate cells (1). We now demonstrate that the opal codon also serves a mosquito-specific function by enabling alphaviruses to avoid triggering an antiviral siRNA response (Figure 7). It may also explain why the opal codon is strictly retained across insect-restricted alphaviruses that do not face any vertebrate- or temperature-specific constraints described in our previous study (1). In light of our current model, it also appears that compromised replication spherule integrity in SINV 550C may lead to increased recognition by the cytoplasmic RNA sensor MDA5 in mammalian cells, resulting in increased IFN induction (Figure 6B) (46).

Opal-to-sense mutations are extremely rare in SINV strains. To date, only the AR86 strain of SINV-like virus has been shown to carry an Opal-to-Cys mutation, which co-occurs with a non-synonymous mutation (nsP1 I538T) near the P1/2 cleavage site (23). Previous studies have demonstrated that this co-occurring nsP1 I538T mutation helps suppress the vertebrate Type I IFN response through STAT1 inactivation, which contributes to its neurovirulence (60, 61). We recently demonstrated that the SINV I538T mutation helps restore the disruption in nsP processing cadence caused by the Opal-to-Cys mutation by a ‘double delay’ (1). However, such a ‘double delay’ would not be predicted to ameliorate the spherule integrity defects. Our observation that SINV 550C also induces a higher IFN response suggests the intriguing possibility that the nsP1 I538T mutation in SINV AR86 may have arisen to help counteract the elevated IFN response induced via the Opal-to-Cys mutation.

One of the central conflicts faced by viruses infecting evolutionarily divergent host species is that adaptations that provide fitness benefits in one host, such as vertebrates, may prove deleterious in another host, such as mosquitoes (62–68). Other viral adaptations might confer benefits in both hosts. Our study highlights one adaptation – the nsP3 opal codon – that provides a benefit in both hosts by maintaining replication spherule integrity, thereby protecting viral RNA from cytoplasmic nucleases and sensors in both insect and vertebrate hosts (Figure 7). In so doing, this single codon confers a fitness advantage in RNAi-competent mosquito cells while also limiting interferon responses in mammalian cells. Thus, the retention of the opal codon by alphaviruses reflects evolutionary pressure from both vector and vertebrate immunity. By reframing the alphavirus opal codon as a multipotent defensive adaptation, our work reframes a classical paradigm of alphavirus biology, while opening new avenues for research into vector-borne disease control.

## Materials and Methods

### Insect and Mammalian Cell Culture

C6/36 *Aedes albopictus* cells were grown at 28°C under 5% CO_2_ in humidified incubators and were cultured in high-glucose, L-glutamine Minimal Essential Medium (Gibco) supplemented with 10% fetal bovine serum (Cytiva) and 1% penicillin-streptomycin (Gibco). U4.4 *Aedes albopictus* cells were grown at 28°C under 5% CO_2_ in humidified incubators. They were cultured in high-glucose, L-glutamine Mitsuhashi and Maramorosch Insect Medium w/o Sodium bicarbonate (VWR) supplemented with 0.12gm/L Sodium bicarbonate, 10% fetal bovine serum (Cytiva), and 1% penicillin-streptomycin (Gibco).

### CRISPR editing of Dicer 2 in U4.4 cells

5×10^5^ U4.4 cells seeded into 6-well plates were co-transfected with 6.25 μg purified SpyCas9-NLS (PNA Bio #CP01–50) and synthesized single guide RNA (sgRNA) (AUAUUCGACGAAUGUCACCA) targeting the 5’ end of the *Dcr2* gene (Synthego). Guide RNA was designed using CHOP-CHOP and verified using Cas-OFFinder to determine off-target effects by searching the guide RNA sequence against the *Aedes albopictus* genome (JXUM01). Synthesized sgRNA were also modified with 5’ 2’-O-methyl (OMe) analogs and 3’ phosphorothioate (PS) to enhance stability and performance. We harvested 1×10^6^ cells two weeks following Cas9-sgRNA transfection, extracted gDNA, and amplified the sgRNA target region using flanking primers (Fwd: 5’-GACCCATATCGCCCTTATGGC-3’, Rev: 5’-TCGTAACTCCCAACGGTGGC-3’). Purified PCR products were sent for Sanger sequencing, and the resulting chromatograms were used for TIDE analyses (version 5.0), which revealed a knockout efficiency of 82–85% (Figure S2) (69).

### Ago2 silencing in U4.4 cells

We used dsRNA to silence *Aedes albopictus Ago2* in U4.4 cells using previously described methods (42). Briefly, total RNA extracted from U4.4 cells was used to generate cDNA using oligo(dT). The cDNA was used as the template for PCR to amplify a region within the PIWI domain of *Ago2* using primers carrying T7 promoters at the 5’ end (Fwd: 5’-TAATACGACTCACTATAGCATCGAGGGTTTCCGCTACA-3’, Rev: 5’-TAATACGACTCACTATAGGGTAATATGTCAGCGCCTGC-3’). Purified PCR products were used as templates for *in vitro* transcription using T7 RNA polymerase (NEB) to generate double-stranded RNA (dsRNA), which was then purified using the MEGAclear clean-up kit (Invitrogen) according to the manufacturer’s instructions. 5×10^5^ U4.4 cells were transfected with 500ng *Ago2* dsRNA using Lipofectamine 3000 (Invitrogen) before assessing *Ago2* silencing over 1–4 days using qRT-PCR (Fwd: 5’- ACTGGATAAGAAACGGGGCG-3’, Rev: 5’-TGCTTGGCGACGATGTTTTG-3’). To determine the extent of functional disruption of the siRNA pathway following *Ago2* silencing, we used a previously described eGFP-reporter silencing assay via dsRNA targeting eGFP (dsGFP) (Figure S5A). Infections were performed 48 hours post *Ago2* dsRNA treatment.

### Small RNA sequencing and analysis

10 μg of total RNA was isolated from cultured U4.4 and C6/36 mosquito cells five days following infection with SINV. RNA purity and integrity were analyzed using Agilent 4200 TapeStation. All subsequent small RNA enrichment steps were performed using 10 μg of total RNA by Novogene, USA. Small RNA sequencing was conducted using an Illumina Novaseq (Novogene, USA). Three biological replicate libraries were sequenced for each sample. After the removal of adaptor sequences (Trimmomatic), processed reads were mapped to the SINV genome (strain TE12) using Geneious Mapper with a mapping quality set to 30, 10% gap, and 20% mismatch rate (). The standard counts-per-million (CPM) method ((CPM = gene read count / total mapped reads) × 1,000,000) was used to normalize for sequencing depth for comparing small RNA counts between samples. Logoplots showing nucleotide frequencies within vpiRNA reads were generated using 24–31nt vpiRNA reads and visualized using WebLogo 3 (70).

### Virus Constructs

All viral stop codon mutant plasmids were generated via site-directed mutagenesis. SINV sense-codon variants were generated in our previous study (1). Mutagenic primers were designed using Takara’s In-Fusion Cloning Primer Design tool. PCR reactions were performed using Phusion polymerase (NEB) with the following cycling conditions: 1. 98°C for 30 sec, 2. 98°C for 30 sec, 3. 50°C for 30 sec, 4. 72°C for 7 min (Repeat steps 2–4 18x), 5. 72°C for 10 min, 6. Hold at 4°C. Reactions were then treated with DpnI for 6 hours and purified using the Monarch PCR and DNA Clean-up Kit (NEB) according to the manufacturer’s protocol. The purified reaction was then transformed into DH5α cells, and DNA was isolated using the Monarch Plasmid Miniprep Kit (NEB) according to the manufacturer’s protocol. (see Suppl Table 1 for specific protocols). Luciferase-based reporter viruses were generated by digesting parental and mutant plasmids with *SpeI* (NEB) and cloning in luciferase (Nano) using HiFi Gibson assembly (NEB). The Gibson reactions were then transformed into DH5α cells, and DNA was isolated using the Monarch Plasmid Miniprep Kit (NEB) according to the manufacturer’s protocol. (see Suppl. Table 1 for specific protocols and primers.

### Independent Viral Growth Assays

2 μg of infectious clones were linearized with *XhoI* (NEB) and subjected to *in vitro* transcription (IVT) using SP6 RNA polymerase (NEB) according to the manufacturer’s suggested protocol. Cells were seeded into 24-well plates to achieve 70–80% confluency and transfected with IVTs using Lipofectamine LTX (Thermo Fisher) according to the manufacturer’s protocol. Cells were collected 48 hours post-transfection and analyzed by flow cytometry (BD Fortessa). Infection rates of all variants were normalized relative to that of wild-type SINV (550opal).

### Viral Competition Assays

Genome copies of WT and mutant virus stocks were calculated using quantitative RT-PCR. Briefly, cDNA synthesis was performed on the viral supernatant using M-MuLV Reverse Transcriptase (NEB) with oligo(dT) (20mer + 5’-Phos) (IDT) according to the manufacturer’s protocol. RT-qPCR analysis was performed using SYBR Green master mix (Thermo Fisher) with gene-specific primers, according to the manufacturer’s protocol, on the Applied Biosystems StepOnePlus Real-Time PCR System (Life Technologies). Once obtained, WT and mutant SINV virus stocks were normalized to equal genome copies. Wild-type and *Dcr2* KO U4.4 cells, and C6/36 cells were seeded into 24 wells and infected with TE3’2J-mCherry wild-type virus alongside TE3’2J: eGFP wild-type or mutant viruses at a 1:1 ratio (MOI – 0.1). Cells were collected 48 hours post-infection and analyzed by flow cytometry (BD Fortessa) to determine the percentage of cells infected with wild-type (red) and variant (green) viruses. Viral competitive index was calculated as the ratio of variant (eGFP) versus wild-type (mCherry) infected cells within the population (71).

### Generation of trans-heterozygous Dcr2 mutant mosquitoes

#### Dcr-2 eGFP null line:

We previously developed a transgenic line containing eGFP, which was originally described in Basu et al. (25). We initially created an out-of-frame deletion using TALEN technology. Loss-of-function was confirmed through multiple approaches, including crossing into our “sensor” line, and observation of disease phenotypes following challenge with both alphavirus and flavivirus pathogens (26, 72). Using the same TALEN target site, we knocked in a construct containing eGFP under the control of the Poly-UB promoter, which is also described in Basu et al (25).

#### Dcr-2 dsRED null line:

For insertion of the dsRED-containing construct, we employed sgRNA-directed Cas9 nuclease. The sgRNA was designed to target a site adjacent to the original TALEN location. We developed two distinct donor constructs featuring different homology arm sequences. Prior to designing these constructs, we sequenced the target site, which revealed polymorphisms within the intronic regions of the homology arms. To account for this genetic variation, we generated donor constructs based on the two most prevalent homology arm sequences identified across individual mosquito specimens. Both donor constructs were co-injected during the transformation procedure. We confirmed one insertion site junction, but the second junction remains unverified. However, confirming genomic insertions in *Ae. aegypti* presents significant technical challenges due to the genome’s architecture and heterogeneity, particularly the presence of numerous large introns containing many repetitive sequences, and the previously mentioned polymorphisms.

#### *Dcr2* double knockouts:

We generated trans-heterozygous *Dcr2*−/− mosquitoes by crossing the two distinct Dcr-2 mutant lines, one expressing eGFP (whole body expression) and the other dsRED (whole body expression) (Figure 2A). The progeny from these crosses were screened at the larval stage for fluorescent marker expression.

### In vivo mosquito infections

Adult *Aedes aegypti* mosquitoes were infected by injecting 0.5 μl of Sindbis virus (SINV; 10^6^ TCID_50_/ml) diluted in Dulbecco’s Modified Eagle Medium (DMEM) directly into the thorax. Wild-type siblings, identified as negative for EGFP and dsRED fluorescence, were used as controls. Following inoculation, *Dcr2* null (*Dcr2* −/−) mutants and wild-type siblings were maintained at 28 °C and 80% relative humidity under a 14:10 h light: dark cycle. Mosquitoes were collected at 0 and 72 hours post-infection in pools of five, flash frozen in liquid nitrogen, and stored at −80 °C until further analysis. Quantitative RT-PCR (qRT-PCR) analysis was carried out to measure viral RNA levels in total RNA extracted from pools of four mosquitoes per replicate.

### Total RNA Extractions and Real-Time Quantitative RT-PCR Analysis

To quantify the copies of minus-strand and plus-strand RNA, cells were seeded into a 24-well plate and infected with WT or variants at greater than 90% confluency. At 4 and 18 hours post-infection, cells were harvested using TRIZOL reagent (Thermo-Fisher), and RNA was extracted using the Direct-zol RNA Miniprep kit (Zymo) according to the manufacturer’s protocol. Following RNA extraction, cDNA was synthesized using M-MuLV Reverse Transcriptase (NEB) with strand-specific primers or OligoDT (20mer+5’Phos) (IDT) according to the manufacturer’s protocol. In all cases, RT-qPCR analysis was performed using SYBR Green master mix (Thermo Fisher) with plus-strand-specific primers according to the manufacturer’s protocol, and the Applied Biosystems StepOnePlus qRT-PCR machine (Life Technologies) was used.

### Viral Replication Assays

SINV nsP3-Nluc reporter viruses (Table S1) were generated by digesting 2μg plasmid DNA with *XhoI* and performing in vitro transcription (IVT) using SP6 RNA polymerase (NEB) according to the manufacturer’s protocol. Cells were seeded into black-walled, clear-bottom 96-well plates at 70–80% confluency and transfected with 100 ng of capped viral RNA using Lipofectamine LTX (Thermo-Fisher) according to the manufacturer’s protocol. At 48 hours post-transfection, translation was quantified using the NanoGlo luciferase assay system (Promega) according to the manufacturer’s protocol. Luminescence was recorded using a Cytation3 Imaging Reader (BioTek).

### Western Blot Analyses

Cells were seeded into six-well plates and infected at greater than 90% confluency (MOI = 5). Cells were washed with cold 1X PBS, and lysates were harvested using RIPA buffer (Pierce) supplemented with 1X protease inhibitor (cOmplete) after a 1-hour incubation at 4 °C. Purified protein was denatured using 2X Laemmli buffer (BioRad) with 5% β-Mercaptoethanol (Sigma-Aldrich). Western blots were run on Mini-PROTEAN precast gels and then transferred to a Trans-Blot 0.2 μm nitrocellulose membrane using the Trans-Blot Turbo Transfer System (Bio-Rad). Blots were incubated in 5% BSA and primary α-FLAG antibody (Proteintech) at 1:3,000, α β-actin antibody (Abcam) at 1:3,000 dilution overnight at 4°C, and washed with 1X TBS with 0.1% Tween-20. The blots were then probed with a secondary α-rabbit HRP-conjugate (R&D Systems). Blots were visualized using a Bio-Rad ChemiDoc Imaging System. Western blots probing for non-structural protein expression and polyprotein processing were performed with independent biological replicates for each virus under different host conditions.

### Immunofluorescence Confocal Microscopy

Glass-bottom #1.5 coverslip 12-well plates (NC0190134; Thermo Fisher Scientific) were treated with 200 μl of sterile 0.01% poly-L-lysine for cell culture (P4832; Sigma-Aldrich) per well for 1 hour at room temperature, rinsed once with DPBS++ (14-040-216; Thermo Fisher Scientific), and allowed to dry for 1 hour. U4.4 cells were seeded onto poly-L-lysine-coated wells and grown to approximately 20% confluency before infection with wild-type (WT) or 550C SINV 3XF-GFP at high multiplicity of infection (MOI) for 48 hours. Cells were rinsed twice with DPBS++ and then fixed with 200 μl of 4% (v/v) paraformaldehyde (28906; Thermo Fisher Scientific) in DPBS++ for 10 minutes, followed by two washes. Permeabilization was performed using 400 μL of 0.05% Triton X-100 (BP151; Thermo Fisher Scientific) in DPBS++ for 5 minutes, followed by two washes. Cells were then blocked with 400 μl of 10% (w/v) bovine serum albumin (BSA; BP9703100; Thermo Fisher Scientific) in DPBS++ for 1 hour.

The following steps were carried out in the dark to minimize photobleaching. Primary antibody staining was performed using 500 μl of mouse monoclonal anti-dsRNA J2 (1:1000) (76651L; Cell Signaling) in 5% BSA, incubated overnight at 4°C with gentle agitation. Cells were washed three times with DPBS++ and blocked again with 400 μl of 10% BSA in DPBS++ for 1 hour. Secondary antibody staining was performed using 500 μl of Alexa Fluor 633-conjugated goat anti-mouse antibody (1:1000) (A-21050; Invitrogen) in 5% BSA, incubated overnight at 4°C without agitation. After washing once with DPBS++, the nuclei were counterstained with Hoechst 33258 (1:1000) (H1398; Invitrogen) in 5% BSA for 15 minutes and then washed three times. Coverslips were mounted using 150 μl of SlowFade^™^ Diamond Antifade Mountant (S36972; Thermo Fisher Scientific). All steps were carried out at room temperature unless otherwise noted. Each wash used DPBS++ for 5 minutes. Imaging was performed using a Leica STELLARIS confocal microscope (DMi8 stand) with a 63x oil-immersion objective and Leica Application Suite X (LAS X) software. All compared microscopy images were acquired on the same day with the same microscopy settings.

### Microscopy Imaging Analysis

Images were analyzed using FIJI/ImageJ 1.54f with Java 1.8.0_322 (64-bit) and the ImageJ 3D Suite MCIB V3.96 (73–75). Spherule boundaries were determined via MaxEntropy thresholding of anti-dsRNA Alexa Fluor 633 signal and used to create 3D regions of interest (ROIs). The accessibility of spherules to cytoplasmic eGFP was determined by quantifying the mean eGFP signal intensity in entire spherules using the 3D Manager Quantif3D function in the ImageJ 3D Suite plugin. Spherule boundaries (Figure S12D) were determined via MaxEntropy thresholding of anti-dsRNA Alexa Fluor 633 signal and used to create 3D regions of interest (ROIs). Reported mean eGFP signal intensities are on a per-spherule basis. Cellular dsRNA levels were determined by quantifying the mean anti-dsRNA Alexa Fluor 633 signal using the 3D Manager Quantif3D function in the ImageJ 3D Suite plugin. Infected cells were identified by the cytoplasmic expression of eGFP and the presence of spherules. Cellular boundaries were determined via Otsu thresholding of cytoplasmic eGFP signal, and areas excluding eGFP were included using the binary ‘Fill in Holes’ function, creating 3D ROIs as defined by the 3D Object Counter with a minimum threshold of 1,500 cubic voxels. If needed, neighboring cells were separated across the entire stack before quantification using the 3D Watershed function in the ImageJ 3D Suite plugin. Nuclei were used as seeds. Reported mean J2 signal intensities are on a per-cell basis.

### IFN reporter activity

To quantify levels of secreted IFN from infected wild-type or MDA5 KO A549 cells, cell supernatants from wild-type SINV (550opal) and 550C infected cells were harvested 16 hours post-infection and transferred onto 5xISGF3-GLuc Huh7 reporter cells (44). Reporter cells were then incubated at 37°C and 5% CO_2_ for 18 hours to allow GLuc secretion into the media. Secreted GLuc was quantified using Pierce Gaussia Luciferase Assay kit (Thermo Fisher Scientific) on a Cytation3 plate reader (BioTek) as per the manufacturer’s protocol.

### Phylogenetics and RNA Structure Analysis

A multiple sequence alignment of nsP4 coding sequences from 49 extant alphavirus species was constructed using Clustal Omega. Alignments were manually curated using Geneious and maximum likelihood phylogenetic trees, generated using the HKY85 substitution model in PHYML, using 100 bootstrap replicates for statistical support. The phylogenetic tree was visualized using FigTree. RNA secondary structure conservation was determined using RNAalifold, which was performed on a multiple sequence alignment of the 45-nt-long E1-hs region from 2,086 alphavirus sequences collected from BV-BRC (v3.51.7) (76). SHAPE-constrained RNA secondary structure was generated using Vienna RNAfold with SHAPE-MaP data previously generated by *Kutchko et al*. (36).

### Statistics

Statistical analyses were conducted using GraphPad Prism (v10.2, GraphPad Software Inc., San Diego, CA). Data were first checked for a normal (Gaussian) distribution using the Kolmogorov-Smirnov test. For data meeting normality (α = 0.05) and similarity of variance criteria, means were compared using, unless otherwise stated, Two-way ANOVA (for multiple groups with two variables) with post hoc tests used for multiple comparisons. To determine the genetic relationship between viral mutations, we employed a modified Combination Index (CI) analysis based on the Chou-Talalay method to quantify combinatorial effects (77).

## Supplementary Material

Supplement 1

**List of Supplementary Materials:** Figures S1–S12

## Data Availability

All data needed to evaluate the conclusions in the paper are present in the paper and/or the Supplementary Materials. The data can also be found in the following public repository X.
