# Supplementary material for: The Sindbis virus nsP3 opal codon protects viral RNA and fitness by maintaining replication spherule integrity
Source: bioRxiv. 2025 Oct 6:2025.09.27.679005. Originally published 2025 Sep 28. Preprint. [Version 2] doi: 10.1101/2025.09.27.679005 (PMC12485792; doi:10.1101/2025.09.27.679005)
Supplement: Supplement 1 [file media-1.pdf]

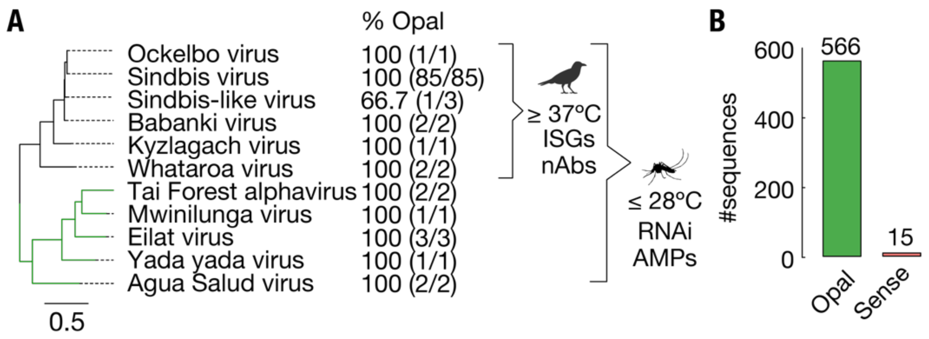

Figure S1. Conservation of the nsP3 opal stop codon among mosquito-isolated alphaviruses. (A) A maximum likelihood phylogenetic tree of two closely related dual-host and insect-specific alphavirus clades was constructed using whole genome sequences. The percent occurrence of the nsP3 opal codon shown next to each taxon was calculated based on publicly available sequences. Note, however, that except for Sindbis virus, which has 85 reported sequences, there are only a few sequences available (shown in parentheses) for each of the other viruses. (B) Percent occurrence of the nsP3 opal codon and the extended PRT codon context UGAC among all available mosquito-isolated alphavirus sequences (n=581). Numbers on top of each bar show the number of analyzed sequences.

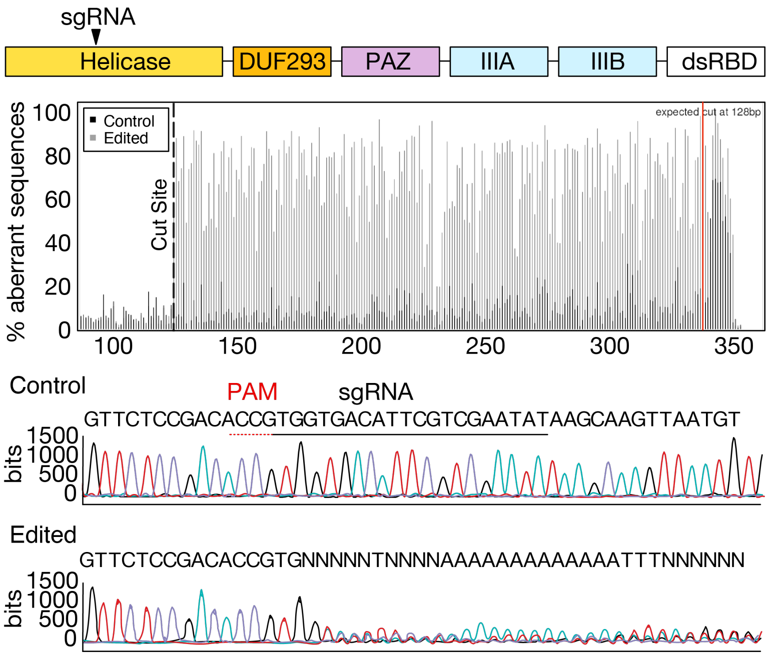

Figure S2. Editing efficiency of *Dcr2* KO U4.4 cells. Domain structure of *Aedes* Dicer 2 protein. The black arrow indicates the approximate location of the CRISPR sgRNA target. (Middle) TIDE analysis plot showing editing efficiency in U4.4 cells. The vertical dashed line represents the expected cut site. (Bottom) Chromatogram of the *Dcr-2* target region in control and CRISPR-edited cells. The region past the vertical red line shows a drop in sequence quality.

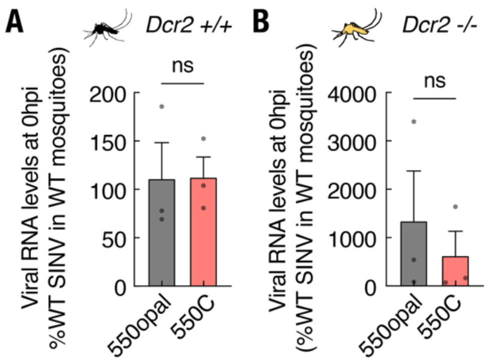

Figure S3. Initial viral inoculum levels among injected mosquitoes. (A) Wild-type (*Dcr2 +/+*) and (B) *Dcr2* KO (*Dcr2 -/-*) *Aedes aegypti* mosquitoes were infected with SINV 550opal and 550C. Approximately 0-2h after infection, injected mosquitoes were collected, and viral RNA was quantified via qRT-PCR. Data represent three independent biological replicates, consisting of four pooled mosquitoes each. Error bars represent the standard error of the mean (SEM). Unpaired t-test. ns = not significant.

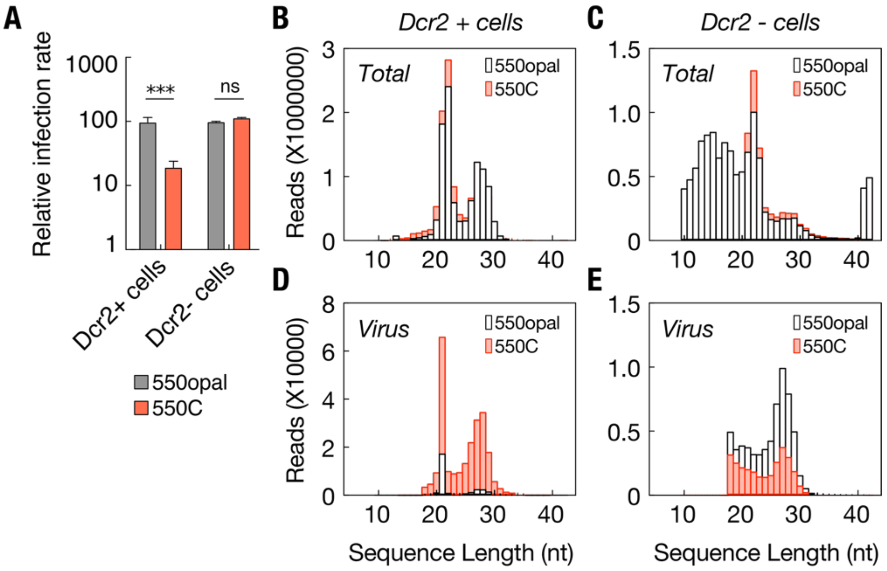

Figure S4. Small RNA read distribution in *Dcr2*+ U4.4 and *Dcr2*- C6/36 cells. (A) Relative infection rates of SINV 550opal and 550C in *Dcr2*+ and *Dcr2*- cells used to extract small RNA. (B-C) Total read counts of small RNA isolated from *Dcr2*+ (B) and *Dcr2*- (C) cells infected with wild-type SINV 550opal and SINV 550C. (D-E) Virus-specific read counts of small RNA isolated from *Dcr2*+ (D) and *Dcr2*- (E) cells infected with wild-type SINV 550opal and SINV 550C. The data represent three independent biological replicates. Error bars represent the standard error of the mean (SEM). Two-way ANOVA with Tukey's multiple comparisons test. *** = P<0.001, ns = not significant.

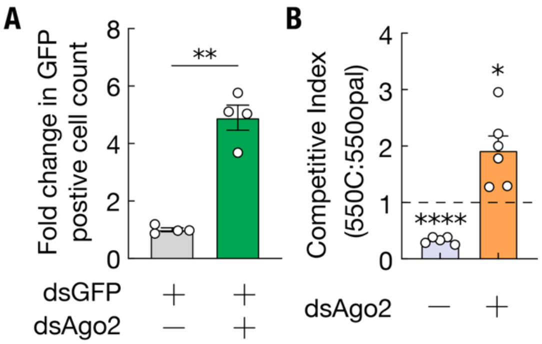

Figure S5. Effect of *Ago2* KD on siRNA activity and SINV 550C competitive fitness in *Dcr2*+ cells. (A) Effect of dsRNA-mediated *Ago2* silencing on GFP reporter expression in *Dcr2*+ mosquito cells co-treated with *GFP* double-stranded RNA (dsGFP) as quantified by flow-cytometry. Error bars represent the standard error of the mean (SEM). Student's t-test. ** = P > 0.01. Two-way ANOVA with Tukey's multiple comparisons test. (B) Effect of dsAgo2 treatment on the replication of wild-type SINV 550opal and SINV 550C in *Dcr2*+ mosquito cells as quantified by luciferase assay. The data is representative of two independent experiments. (B) Competitive fitness of SINV 550C variant against wild-type SINV 550opal in U4.4 cells treated with dsAgo2. The data represent five independent biological replicates. Error bars represent the standard error of the mean (SEM). One-sample t-test compared to an neutral competitive index (550C:550opal) of 1. **** = P < 0.0001, * = P < 0.05.

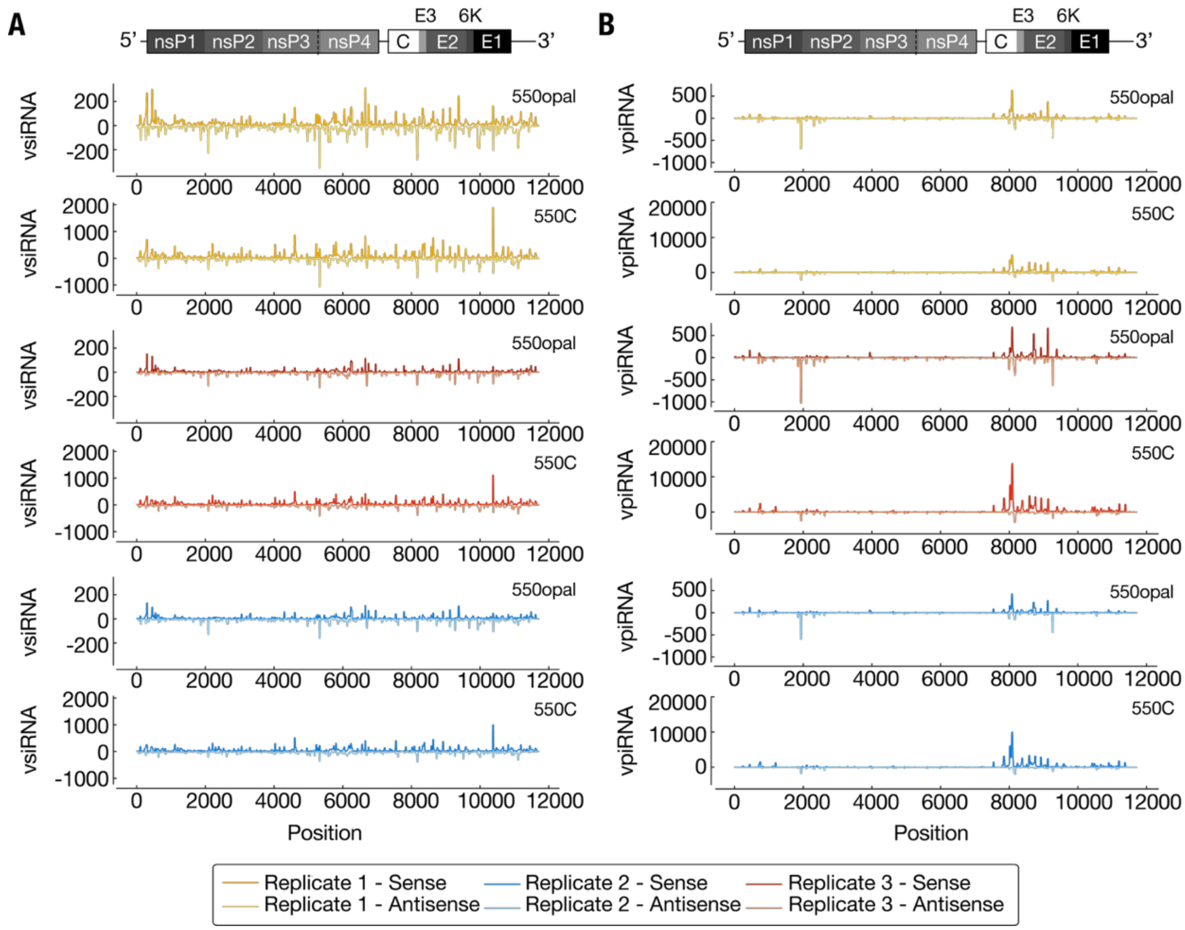

Figure S6. Replicate data for virus-mapped vsiRNA and vpiRNAs in *Dcr2*+ mosquito cells. (A) Normalized vsiRNA read distribution in *Dcr2*+ cells infected with wild-type SINV 550opal and SINV 550C. (B) Normalized vpiRNA read distribution in *Dcr2*+ cells infected with wild-type SINV 550opal and SINV 550C.

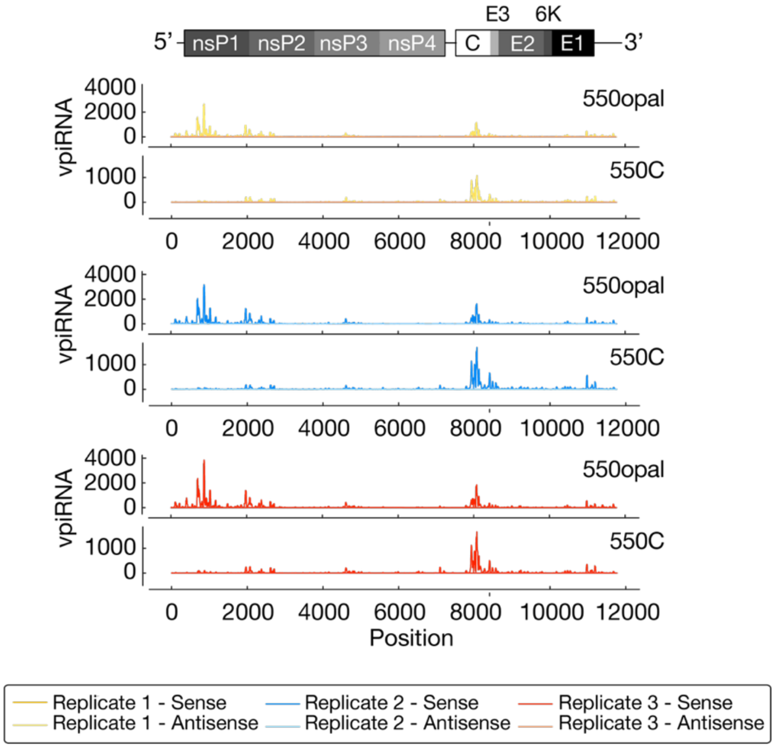

Figure S7. Replicate data for virus-mapped vpiRNAs in *Dcr2*– mosquito cells. Normalized vpiRNA read distribution in *Dcr2*– cells infected with wild-type SINV 550opal and SINV 550C.

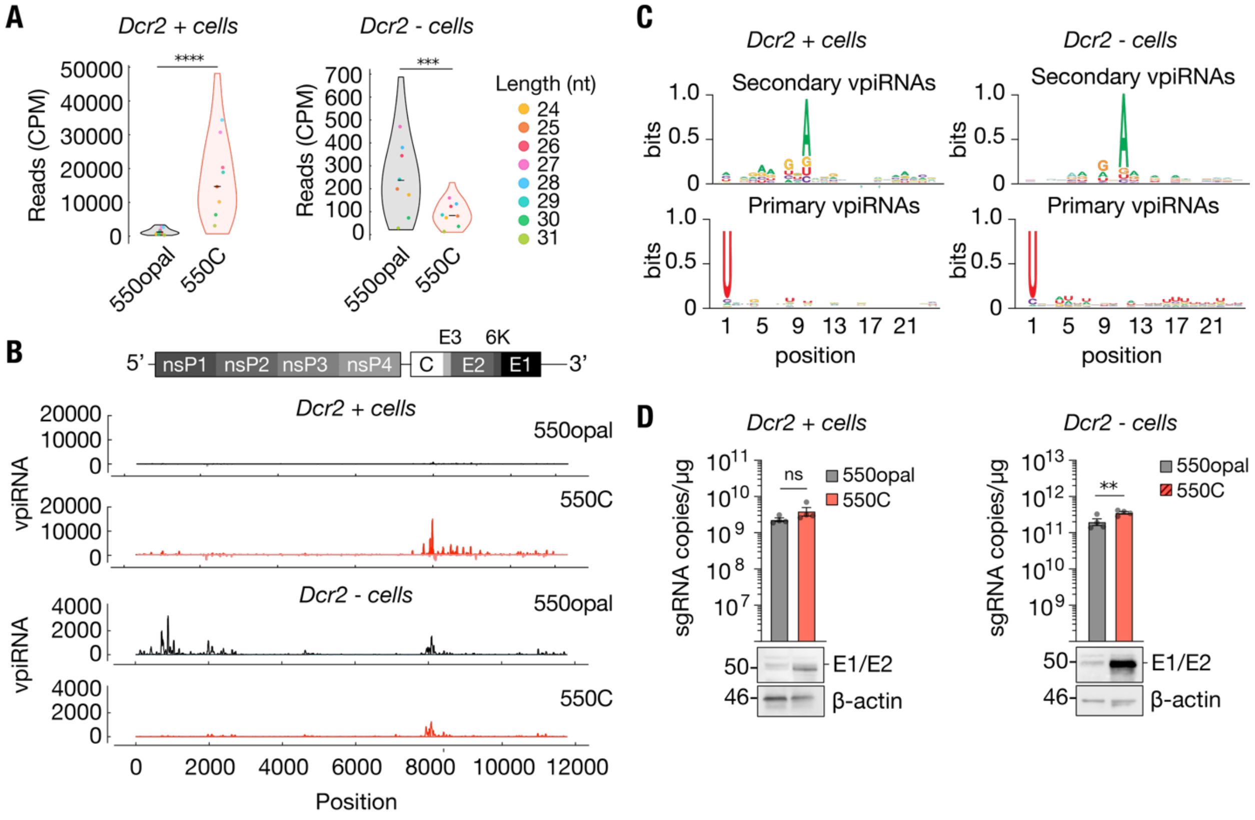

Figure S8. Virus-derived piRNAs are not antiviral in mosquito cells. (A) Normalized median read counts of vpiRNAs of different size classes in *Dcr2*+ U4.4 cells infected with wild-type SINV 550opal or SINV 550C viruses. Student's t-test. (B) Distribution of vpiRNA reads derived from wild-type SINV 550opal (in grey) or SINV 550C (in red) viruses in *Dcr2*+ U4.4 cells. Positive and negative Y-axis values represent read counts mapped to the sense and antisense strands at every position along the SINV genome and subgenome (X-axis). (C) Cumulative per-position nucleotide frequency of the first 24 bases in sense (Top) and antisense (Bottom) vpiRNA reads derived from *Dcr2*+ U4.4 cells infected with wild-type SINV 550opal or SINV 550C viruses. Logoplots were generated using WebLogo 3. (D) (Top) Levels of intracellular viral subgenomic RNA in *Dcr2*+ U4.4 cells infected with wild-type SINV 550opal or SINV 550C viruses as quantified via qRT-PCR. (Bottom) Western blot analysis of E1/E2 structural glycoprotein levels in *Dcr2*+ U4.4 cells infected with wild-type SINV 550opal or SINV 550C viruses. Data is representative of four independent biological replicates. Error bars represent the standard error of the mean (SEM). Student's t-test. **** = P < 0.0001, ns = not significant.

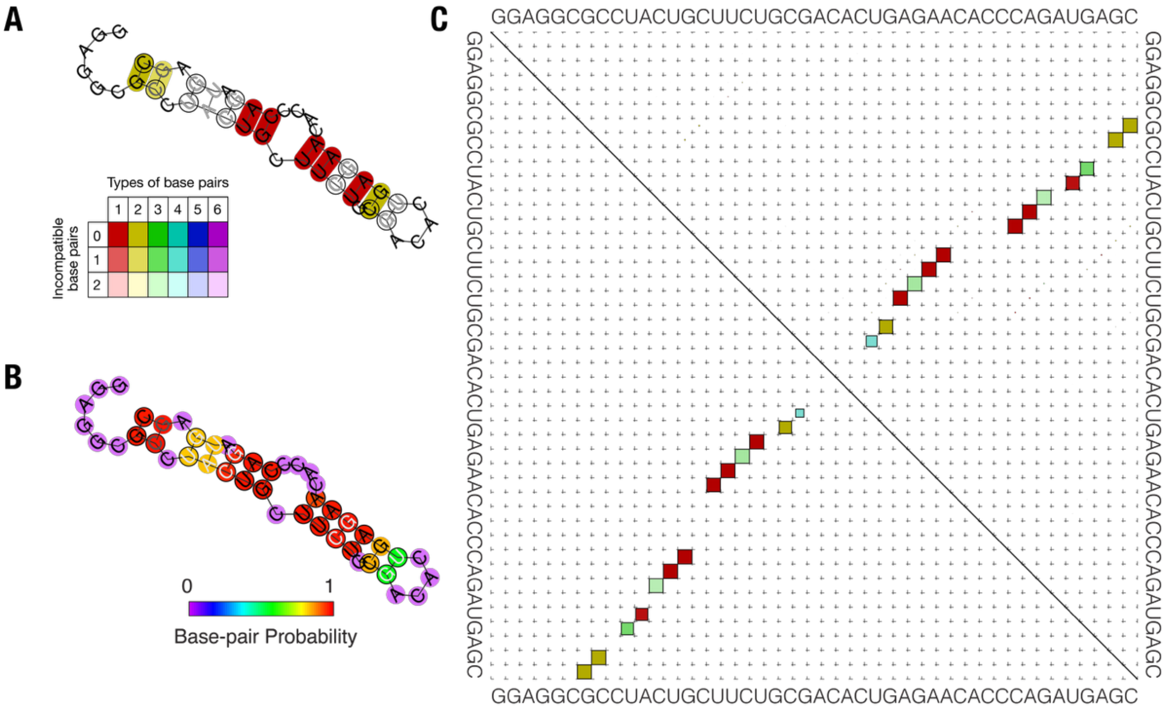

Figure S9. Sequence and structural conservation of E1-hs RNA across alphaviruses. (A) RNAlifold predicted structure of the E1-hs region spanning SINV nucleotides 10328-10372. Colors highlight the mutational pattern with respect to the predicted RNA structure. The color key indicates whether one predicted base pair is formed by several different combinations of nucleotides, consistent or compensatory mutations have occurred. Pale colors indicate the absence of base-pairing in some sequences of the alignment. (B) Base-pairing probability of the predicted RNA structure. (C) Base-pairing probability matrix of the E1-hs region. The upper triangle shows pairing probabilities, with box size proportional to the likelihood of a base pair (i, j). The lower triangle depicts the minimum free energy (MFE) structure using uniformly sized boxes. In alignments, dot plots are color-coded to indicate sequence variation (refer to panel A). Note: Some weakly conserved base pairs are not indicated in panel A but are visible in panel C.

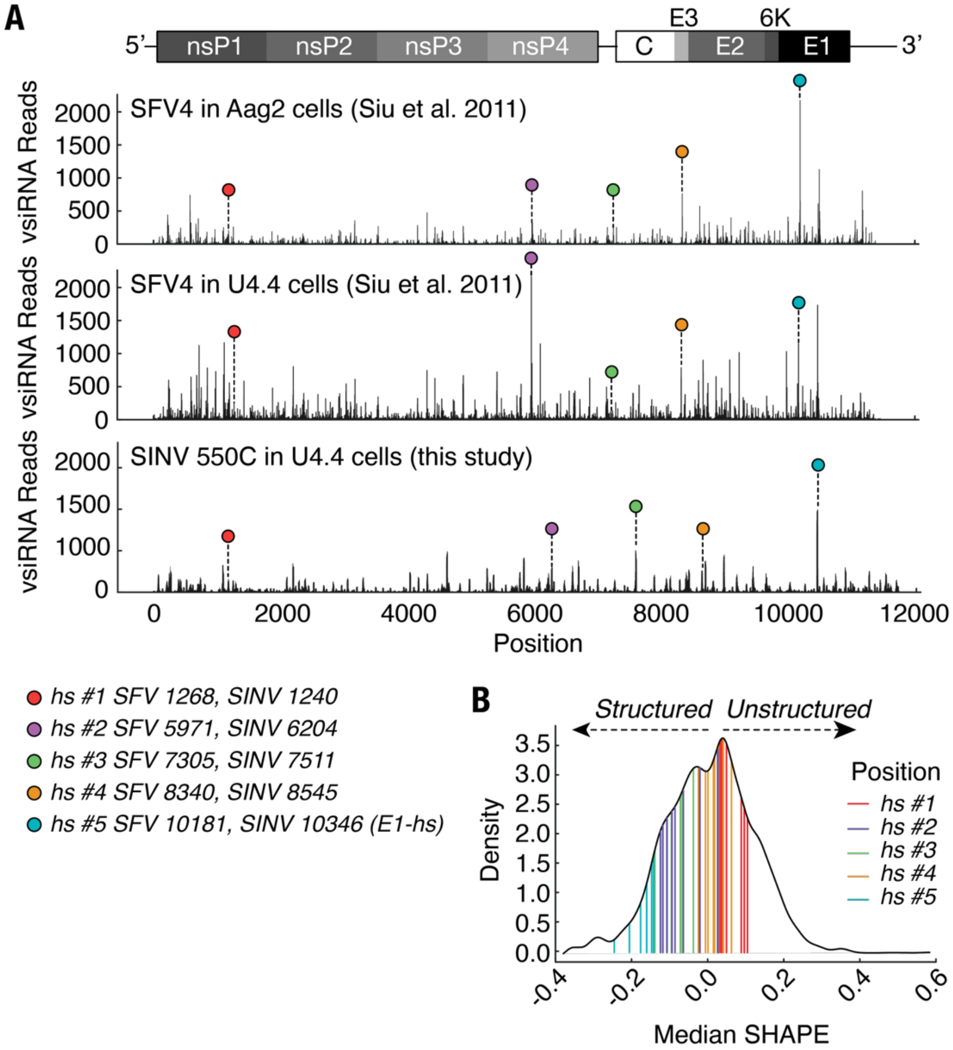

Figure S10. Shared vsiRNA hotspots between distantly related alphaviruses. (A) vsiRNA hotspots identified within the Semliki Forest virus (SFV4) genome by *Siu et al.* in RNAi-competent *Aedes aegypti*-derived Aag2 (Top) and *Aedes albopictus*-derived U4.4 (Middle) cells. (Bottom) vsiRNA hotspots identified within SINV 550C in *Aedes albopictus*-derived U4.4 cells in this study. Four shared hotspots at SINV positions 1240, 7511, 8545, and 10346 are highlighted with colored circles (see key for details). (B) Density distribution profile of median SHAPE values of SINV RNA. Median SHAPE values of hotspot region residues are highlighted in color.

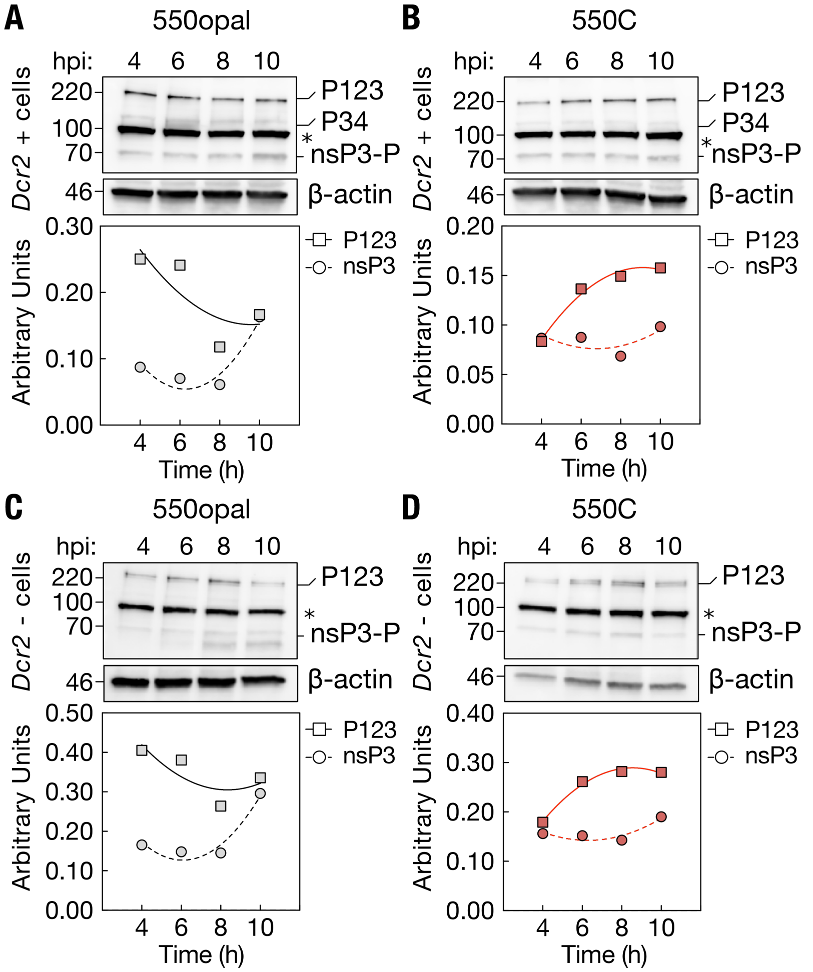

Figure S11. Non-structural polyprotein processing cadence in mosquito cells. Temporal changes in the levels of unprocessed (P123) and processed (nsP3) nsP protein as determined by densitometric analysis of western blot data from (A-B) *Dcr2*+ (U4.4) and (C-D) *Dcr2*– (C6/36) cells infected with wild-type SINV 550opal (A, C) and SINV 550C (B, D). Harvested protein lysates were probed with anti-FLAG (nsP3) antibody. β-actin was used as a loading control. The data is representative of two independent experiments.

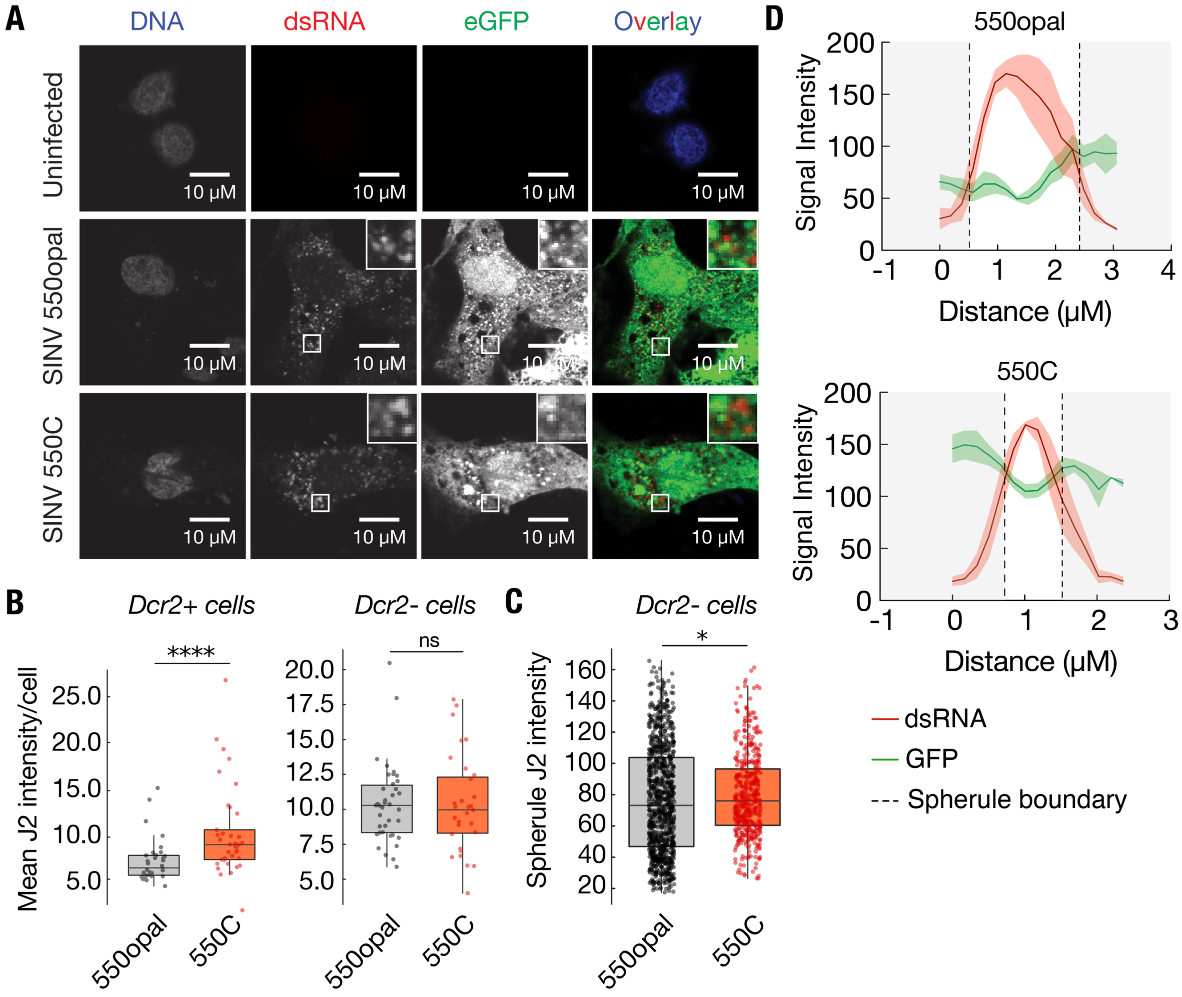

Figure S12. J2 signal quantification in SINV infected *Dcr2-* cells. (A) Localization of viral dsRNA (identified by the J2 antibody) and virally encoded GFP in *Dcr2*- C6/36 cells that were either uninfected or infected with wild-type SINV 550opal (top) or SINV 550C (bottom) as determined by confocal microscopy. The inset shows the localization of GFP and dsRNA around viral replication spherules. (B) Mean J2 intensity per cell in *Dcr2*+ U4.4 (left) and *Dcr2*- C6/36 (right) cells infected with wild-type SINV 550opal and SINV 550C. (C) Spherule J2 intensity in *Dcr2*- C6/36 cells infected with wild-type SINV 550opal and SINV 550C. Error bars indicate the standard error of the mean (SEM). Mann-Whitney U-test.**** = $P < 0.0001$, * = $P < 0.05$, ns = not significant. (D) Two-dimensional (2D) plot profile of spherules in *Dcr2*+ cells infected with wild-type and variant SINV. Dashed vertical lines indicate spherule boundaries.
